# Prevalence of Antimicrobial Resistance and Hemolytic Phenotypes in Culturable Arctic Bacteria

**DOI:** 10.3389/fmicb.2020.00570

**Published:** 2020-04-03

**Authors:** Diana C. Mogrovejo, Laura Perini, Cene Gostinčar, Kristina Sepčić, Martina Turk, Jerneja Ambrožič-Avguštin, Florian H. H. Brill, Nina Gunde-Cimerman

**Affiliations:** ^1^Dr. Brill + Partner GmbH Institut für Hygiene und Mikrobiologie, Hamburg, Germany; ^2^Department of Biology, Biotechnical Faculty, University of Ljubljana, Ljubljana, Slovenia; ^3^Lars Bolund Institute of Regenerative Medicine, Qingdao, China

**Keywords:** Arctic, extremely cold environment, pathogens, hemolysis, antimicrobial resistance

## Abstract

Many Arctic biomes, which are populated with abundant and diverse microbial life, are under threat: climate change and warming temperatures have raised concerns about diversity loss and possible emergence of pathogenic microorganisms. At present, there is little information on the occurrence of Arctic virulence-associated phenotypes. In this study we worked with 118 strains of bacteria (from 10 sampling sites in the Arctic region, located in Greenland and the Svalbard Archipelago) isolated using R2A medium. These strains belong to 4 phyla and represent 36 different bacterial genera. Phenotypic resistance to 8 clinically important antimicrobials (ampicillin, chloramphenicol, ciprofloxacin, cefotaxime, erythromycin, imipenem, kanamycin, and tetracycline) and thermotolerance range were determined. In addition, a screening of all isolates on blood agar media and erythrocytes suspension of bovine and sheep erythrocytes for virulence-linked hemolytic activity was performed. Although antimicrobial resistance profiles varied among the isolates, they were consistent within bacterial families and genera. Interestingly, a high number of isolates (83/104) were resistant to the tested concentration of imipenem (4 mg/L). In addition, one third of the isolates showed hemolytic activity on blood agar, however, in only 5% of the isolates hemolytic activity was also observed in the cell extracts when added to erythrocyte suspensions for 60 min. The observed microbial phenotypes contribute to our understanding of the presence of virulence-associated factors in the Arctic environments, while highlighting the potential risks associated with changes in the polar areas in the light of climate change.

## Introduction

The Arctic region, once considered a “biological desert,” is now regarded as a rich and dynamic ecosystem (Krajick, 2001; Post et al., 2009), characterized by diverse biomes that harbor species from all three domains of life (Anesio and Laybourn-Parry, 2012). Until the onset of global warming, the changes of extreme Arctic biomes progressed slowly, compared to temperate and tropical regions, and served as proxies for unperturbed natural environments. In the last decades, researchers increasingly recognized the opportunities that the Arctic biomes represent for studying of diverse microbial communities and their interactions (Post et al., 2009).

Ongoing climate change is drastically altering our planet, increasing global air and ocean temperatures, causing widespread melting of snow and ice and rising global average sea levels (Intergovernmental Panel on Climate Change [IPCC], 2007). While every environment on Earth is influenced, Arctic environments are particularly affected and, in fact, their average temperatures have increased almost twice as fast as the global warming rate in the past 100 years (Intergovernmental Panel on Climate Change [IPCC], 2007).

Despite the fact that climate change directly affects microorganisms, microbial life is rarely studied in this context (Cavicchioli et al., 2019). When their interactions with other species are perturbed, the resulting pressures strongly influence microbial community composition and function and might play a role in the expression of bacterial virulence factors (Livermore, 2003). Additionally, altered climatic conditions might enable virulent and/or antimicrobial resistant microbes, originating from temperate regions, to reach previously pristine and unperturbed Arctic environments, spreading virulence factors and resistance genes (Altizer et al., 2013).

Antimicrobial resistance is the natural or acquired capacity of microorganisms to withstand the effects of an antimicrobial drug (Davies and Davies, 2010; World Health Organization [WHO], 2014). In fact, resistance to antimicrobials is widely considered to be one of the biggest health challenges of our time, leading to higher medical costs, prolonged hospital stays, and increased mortality rates (World Health Organization [WHO], 2014). Several studies have confirmed the presence of antimicrobial resistance genotypes and phenotypes in microorganisms from cold environments and suggested that the polar regions can be considered benchmarks for the study of antimicrobial resistance in pristine non-clinical environments (Yuan et al., 2014; Tam et al., 2015; Agnew et al., 2016; McCann et al., 2019).

Hemolysins, considered an important virulence factor, are compounds produced by a variety of bacterial species. These compounds are responsible for membrane damage, cell lysis and destruction of neighboring cells and tissues in order to provide nutrients, mainly iron, for the toxin-producing bacteria (Bullen et al., 2005). Iron is an essential element for living organisms as it plays catalytic, regulatory and structural roles in the cell and many authors have also pointed out its important role as a virulence regulator for commensal and pathogenic microorganisms (Messenger and Barclay, 1983; Litwin and Calderwood, 1993; Zughaier and Cornelis, 2018). Even though they are studied as major virulence factors in infection models of laboratory animals (Bhakdi et al., 1994; Bayley, 1997; Tomita and Kamio, 1997) and are commonly associated with pathogenic bacteria, the expression of hemolysins has rarely been assessed in bacterial isolates from environmental samples (Bevivino et al., 2002; González-Rodríguez et al., 2007) and, to the best of our knowledge, very few studies exist about the hemolytic activity of Arctic bacterial species, e.g., Mogrovejo-Arias et al. (2020).

Finally, an important bacterial trait is the microorganism’s ability to grow at 37°C, the optimum temperature for most of human pathogens that allows microbial colonization, and is thus a prerequisite for pathogenic microbe-human interactions (Madigan et al., 2012). Research of Arctic microbes usually uses growth temperatures lower than 37°C, e.g., 4, 15, 20, or 27°C (Wiebe et al., 1992; Amato et al., 2007; Tam et al., 2015) and temperatures above 30°C are rarely used, as these are usually too high for the optimal growth of even the psychrotolerant species (Moyer and Morita, 2007).

Environmental or commensal microorganisms might become pathogenic under stressful conditions since bacterial species respond to environmental stress in a variety of ways, e.g., acceleration of horizontal gene transfer (Marshall et al., 2009). Accordingly, genes encoding traits that occur naturally in the environment, such as antimicrobial resistance and hemolytic activity, can be transferred between habitats by bacteria, bacteriophages or mobile genetic elements, resulting in a global-scale redistribution of resistance and virulence factors/genes (Marshall et al., 2009; Davies and Davies, 2010).

One of the main indirect effects of climate change could be the increase of infectious diseases (Kurane, 2010). The Arctic in this context represents an important source of microorganisms and mobile genetic elements that could transit into more human-associated environments (Edwards, 2015). Thus research on virulence-associated phenotypes in natural, non-clinical environments is necessary to understand the possible implications of the climate changes for public health (Martínez, 2008).

In the present study of bacterial isolates from the Arctic region, we investigated three phenotypes commonly associated with pathogenic bacteria, i.e., growth at 37°C, the hemolytic activity on blood agar plates at different temperatures as well as on erythrocyte suspensions and resistance to 8 different, commonly used and clinically relevant antimicrobials.

## Materials and Methods

### Site and Samples Description

Samplings were performed during 2016 and 2017 summer/winter seasons in the south-western margin of Greenland, the Greenland Ice Sheet, and Svalbard, the areas of Ny-Ålesund and Longyearbyen (Perini et al., 2019a, b; Mogrovejo-Arias et al., 2020). The bacterial composition of these ecosystems has been studied extensively by culture-dependent as well as culture-independent methodologies (Perini et al., 2019a, b; Mogrovejo-Arias et al., 2020).

**FIGURE 1:**
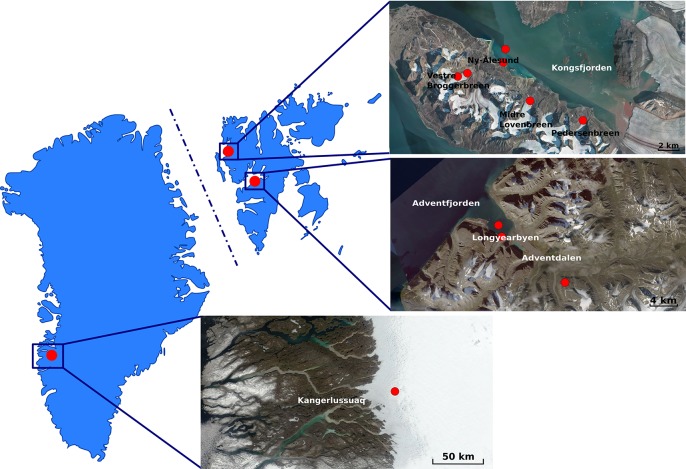
Maps of Greenland **(Left)** and Svalbard **(Right)** Archipelago (Norway) indicating the areas where fieldwork was conducted. Red dots in the satellite images indicate the sampling sites. Greenland map was acquired as an image from Modis Satellite. Svalbard map was adapted as an image from TopoSvalbard credit NPI/USGS Landsat, courtesy of the Norwegian Polar Institute (available at: https://toposvalbard.npolar.no). Greenland and the Svalbard Archipelago are not drawn to scale.

Glacial and subglacial ice, snow, lake ice, tap water, glacial meltwater, sediment, pond and sea water samples were collected from the locations shown in Figure 1 and described in Table 1. Ice, snow, cryoconite, and sediment samples were collected using disposable sterile nitrile gloves and surface-sterilized tools and transferred into sterile Whirl-Pak^®^ plastic bags. For snow and soil samples, 5 cm of the surface were discarded and a disinfected shovel or sterile spoon was used to store the sample in the sterile plastic bags. Tap water, glacial meltwater, pond and sea water were collected into plastic bottles previously washed with 15% hydrogen peroxide, followed by three washes with sterile water and three washes with the sample. Samples were processed as outlined by Mogrovejo-Arias et al. (2020) or processed within 24 h from their collection at the primary ice camp on the Greenland Ice Sheet, the NERC Arctic Research Station (Ny-Ålesund), or the University Centre in Svalbard (UNIS; Longyearbyen).

**TABLE 1:** Sample types and GPS coordinates of the sampling locations for this study.

Sample type	Site description	Area, country	Season	Year	GPS coordinates
Dispersed cryoconite	Ice camp on GrIS	Kangerlussuaq, Greenland	Summer	2016	67°04′43″N 49°20′29″W
Supraglacial ice – clear ice				2016, 2017	
Supraglacial ice with high biomass inclusions of dark glacial algae – dark ice				2016, 2017	
Cryoconite				2016, 2017	
Supraglacial water				2017	
Snow	Ice camp on GrIS	Kangerlussuaq, Greenland	Summer	2017	67°04′43″N 49°20′29″W
	Seasonal pond, Adventdalen	Longyearbyen, Svalbard	Winter	2016	78°09′24″ N 16°01′59″ E
	Midtre Lovénbreen	Ny-Ålesund, Svalbard	Summer	2017	78°53′08″ N 12°02′44″ E
	Vestre Brøggerbreen	Ny-Ålesund, Svalbard	Summer	2017	78°54′42″ N 11°43′42″ E
Subglacial ice	Midtre Lovénbreen	Ny-Ålesund, Svalbard	Summer	2017	78°53′37″ N 12°04′13″ E
	Vestre Brøggerbreen	Ny-Ålesund, Svalbard	Summer	2017	78°54′55″ N 11°45′48″ E
	Pedersenbreen	Ny-Ålesund, Svalbard	Summer	2017	78°52′46″ N 12°17′57″ E
Tap water	UNIS	Longyearbyen, Svalbard	Winter	2016	78°13′21″ N 15°39′06″ E
Glacial melt water	Midtre Lovénbreen	Ny-Ålesund, Svalbard	Summer	2017	78°53′25″ N 12°03′15″ E
					78°53′36″ N 12°04′13″ E
Pond water	Ny-Ålesund area	Ny-Ålesund, Svalbard	Summer	2017	78°55′34″ N 11°56′21″ E
Sea water	Adventfjorden	Longyearbyen, Svalbard	Winter	2016	78°14′27″ N 15°36′59″ E
	Kongsfjorden	Ny-Ålesund, Svalbard	Summer	2017	78°55′33″ N 12°02′29″ E
Soil	Midtre Lovénbreen forefield	Ny-Ålesund, Svalbard	Summer	2017	78°53′54″ N 12°03′59″ E
	Vestre Brøggerbreen forefield	Ny-Ålesund, Svalbard	Summer	2017	78°55′20″ N 11°46′38″ E
Sediment	Marine sediment, Adventfjorden	Longyearbyen, Svalbard	Winter	2016	78°14′27″ N 15°36′59″ E
	Pond sediment, Ny-Ålesund area	Ny-Ålesund, Svalbard	Summer	2017	78°55′34″ N 11°56′21″ E

### Cultivation and Isolation of Bacteria

Snow, supraglacial and subglacial ice were melted at+4°C in the dark prior filtration. After the outer surface layer of subglacial ice was melted, it was discarded, the remaining ice was washed with sterile water and only the second round of meltwater was used for analyses. Serial dilutions were used for soil and sediment samples. Water samples were filtered through Milli-pore membrane filters (0.45 μm pore size) in duplicates. Filters were placed onto R2A (BioLife), an oligotrophic medium for heterotrophic microorganisms.

Plates of all sample types were incubated at 5, 10, 15–17, and 37°C for up to 4 months in sterile plastic bags to preserve the humidity of the agar media. Negative control plates were prepared with sterile water frozen prior to culturing and processed as the samples. Two control agar plates were streaked for each testing temperature and incubated in the same conditions and for the same duration as the samples. Liquid and enrichment cultures were not used.

### Bacterial Identification

Morphologically different colonies were selected and streaked onto fresh R2A plates in order to obtain pure cultures. The plates were incubated at the initial isolation temperature of each isolate and, in addition, at 37°C. DNA was extracted using PrepMan Ultra reagent (Applied Biosystems) according to the manufacturer instructions. 16S rRNA gene was amplified with 27f-lane, 1492R, 8F, and 1541R primers (Tam et al., 2015; Perini et al., 2019a; Mogrovejo-Arias et al., 2020). The 16S nucleotide amplicons were Sanger-sequenced by Microsynth AG (Switzerland). The resulting sequences were analyzed using MUSCLE software (Edgar, 2004) implemented in the MEGA7 package (Kumar et al., 2016) and compared against the GenBank database using the BLAST software.^1^ The 16S rDNA sequences obtained in this study were deposited in the NCBI GenBank nucleotide database. All isolated strains used in this study have been deposited in the Ex Culture Collection of the Infrastructural Centre Mycosmo (MRIC UL) at the Department of Biology, Biotechnical Faculty, University of Ljubljana, Slovenia. Information on all isolates and accession numbers for their 16S rRNA sequences are listed in Supplementary Table S1.

### Hemolytic Assay on Blood Agar

Bacterial isolates were cultured on Blood Agar Base (Fluka Analytical) containing 5% of sterile sheep or bovine blood and incubated at +15 and +37°C for up to 7 days. The use of human blood is generally discouraged (Buxton, 2005) and thus, it was not used in our experiments. The isolates were revived from stock cultures from the Ex Culture Collection of the Infrastructural Centre Mycosmo (MRIC UL), University of Ljubljana. Only actively growing cultures were tested. As controls, the following strains revived from the Ex Culture Collection of the Infrastructural Centre Mycosmo (MRIC UL), University of Ljubljana were used: EXB V-53 *Enterococcus faecalis* (no hemolysis, referred to as γ-hemolysis), EXB V-59 *Streptococcus pyogenes* (β-hemolysis), EXB V-62 *Streptococcus pneumoniae* (α-hemolysis).

### Hemolytic Assay With Erythrocyte Suspension

Hemolytic activity of organic bacterial extracts was tested in real time using bovine and sheep erythrocyte suspensions rather than blood agar. Bacterial cultures (118 environmental and 3 control strains described in Supplementary Table S2) were grown in sterile Falcon tubes containing 20 mL of Nutrient Broth (BioLife). Tubes were shaken at 150 rpm and +15 or +25°C, based on their isolation temperature, for 3 days. Cultures were then centrifuged for 5 min at 12,000 × *g*, supernatant was discarded, and biomass was resuspended in 5 mL of non-denaturated 96% ethanol. Suspensions were vortexed for 5 sec, sonicated (40% amplitude, 20 s) to lyse the cells and shaken (150 rpm) at room temperature for 30 min. Suspensions were subsequently centrifuged (5 min at 12,000 × *g*), the supernatants were transferred into pre-weighted sterile Falcon tubes and the ethanol was evaporated under chemical fume hood in a flow of nitrogen gas. The dry weight of the crude extracts was determined gravimetrically, and the extracts were resuspended in an appropriate amount of non-denaturated 96% ethanol to a standard final concentration of 1 mg extract dry weight/mL.

Hemolytic activity was measured by a turbidimetric method using a microplate VIS absorption reader (Dynex Technologies, United States). Different volumes (0–25 μL) of ethanolic extracts, or of 96% ethanol alone, were pipetted into the wells on the 96-well microtiter plate, and combined with the appropriate volume of erythrocyte buffer (0.13 M NaCl, 0.02 M Tris, pH = 7.4) to obtain different final concentrations of the extract (0–250 μg/mL) in the 100 μL final volume. Right before measurement, 100 μL of bovine or sheep erythrocytes suspension in erythrocyte buffer (OD = 0.5 at 630 nm) was added into the wells. The decrease in apparent absorbance was measured for 60 min, in 30 s intervals, to determine the *t*_50_ (time necessary for 50% hemolysis). The hemolytic activity was expressed as 1/*t*_50_ (min^–1^). When tested alone, ethanol did not induce any visible hemolysis during the 60 min.

### Antimicrobial Susceptibility Test

Bacteria were cultured on Nutrient Agar (BioLife) plates containing 8 different commonly used antimicrobials and incubated at 15 or 25°C, based on the initial isolation temperature of each isolate. Results were observed as soon as the bacterial growth appeared (between 1 and 7 days). The following antimicrobials were used: ampicillin (AMP) 100 mg/L; chloramphenicol (CHL) 25 mg/L; cefotaxime (CTX) 2 mg/L; ciprofloxacin (CIP) 0.25 mg/L; erythromycin (ERY) 15 mg/L; imipenem (IPM) 4 mg/L; kanamycin (KAN) 50 mg/L; tetracycline (TET) 10 mg/L. As positive controls, wild type *Escherichia coli* strains EXB L-4239 A5 and EXB L-4240 A6 isolated from poultry and with known resistance profile were used [Ex Culture Collection of the Infrastructural Centre Mycosmo (MRIC UL), University of Ljubljana]. Imipenem-resistant strains were inoculated on nutrient agar plates with gradually increasing IPM concentrations (4, 6, 8, and 10 mg/L). Strains that showed resistance to higher concentrations were further inoculated in duplicate on CHROMID ^®^ CARBA SMART Agar (BioMérieux, France) and incubated at 15°C to screen for the production of specific carbapenemases: class A *Klebsiella pneumoniae* carbapenemases or KPC; Class B metallo-β-lactamases or MBL including New Delhi metallo-β-lactamases, NDM and Class D OXA-48-like carbapenemases (van Duin and Doi, 2017).

## Results

### Bacterial Isolation and Identification

A total of 290 isolates were obtained from all the samples after incubation (Supplementary Table S1). Nutrient-rich samples, i.e., dark ice, cryoconite, sediment and soil, yielded the majority of isolates (159/290). The isolates belonged to four different phyla: Proteobacteria (104/290), Firmicutes (81/290), Actinobacteria (77/290), and Bacteroidetes (28/290). The most common genera in each phylum were: *Pseudomonas* (39/104), *Bacillus* (49/81), *Cryobacterium* (17/77), and *Flavobacterium* (21/28), respectively. Gram-positive isolates were slightly more frequent (158/290) than Gram-negative (132/290).

Based on 16S rDNA identification, abundance and morphological characteristics, 118 unique strains were selected for further tests (Supplementary Table S2). *Bacillus* sp. and *Pseudomonas* sp. were the most abundant genera in this study (38/118), accounting for more than 30% of the isolates selected for the tests. The majority of the chosen isolates (87/118) were psychrotolerant and were obtained at incubation temperatures of 15–17°C, followed by 17 mesophiles from incubation at 37°C and 14 psychrophiles obtained from incubation at 5–10°C.

Interestingly, 11 isolates obtained at low incubation temperatures (5–10°C) belonging to the genera *Pedobacter* (L-1969 and L-1973), *Sphingomonas* (L-1972), *Raoultella* (L-1980), *Flavobacterium* (L-1981), *Pseudomonas* (L-1983, N40, and N71), *Leifsonia* (An34), *Salinibacterium* (S58), and *Microbacterium* (S60) showed some degree of growth at 37°C on R2A. Out of the 87 psychrotolerant isolates, 74 were able to weakly grow at 37°C. Only 13 psychrotolerant isolates belonging to the genera *Rhodopseudomonas* (L-1894), *Cryobacterium* (L-2263 and L-2279), *Massilia* (L-2271, L-2283, and L-2653), *Flavobacterium* (L-2291 and L-2562), unidentified Oxalobacteraceae (L-2547), *Sphingomonas* (L-2552), *Pseudomonas* (L-2554), *Paenibacillus* (L-2661), and *Streptomyces* (N41) were not able to grow at 37°C.

### Hemolysis on Blood Agar Plates

Two different experiments were performed to observe the hemolytic activity of the isolates. In the first one, the excretion of hemolytically active compounds resulted in either α- or β-hemolysis around the bacterial colonies on blood agar plates. A β-hemolytic reaction implies complete lysis of the red blood cells, causing a clear zone on the agar surrounding the colony and it is referred as true hemolysis. On the other hand, an α-hemolytic reaction occurs when the hemoglobin in the red blood cells is reduced to methemoglobin, causing a greenish discoloration on the agar surrounding the colonies. Finally, the absence of hemolysis or discoloration is referred to as γ-hemolysis (Buxton, 2005).

**FIGURE 2:**
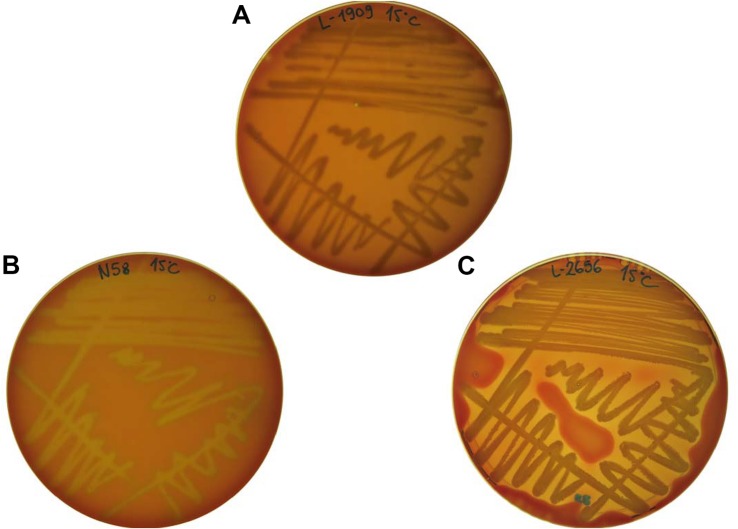
Examples of hemolytic phenotypes expressed by the tested isolates. **(A)** L-1909 showing no hemolysis (referred to as γ-hemolysis); **(B)** N58 showing α-hemolysis; **(C)** L-2656 showing β-hemolysis.

The tested bacterial strains displayed all three phenotypes (Figure 2). Our results showed that the hemolysis was influenced by the incubation temperature (Table 2). *Pseudomonas* spp. showed psychrotolerant behavior, that is, all of them grew at 15°C while only 50% grew at 37°C. Of these, 9/22 isolates were β-hemolytic on both types of blood at 15°C, whereas only 6/22 were hemolytic at 37°C. Isolate L-2644 was the only one that expressed β-hemolysis at both temperatures and on both blood types. No α-hemolytic phenotype was observed for this genus (Table 2).

**TABLE 2:** List of the bacterial isolates tested for thermotolerance, hemolysis on blood agar at 15 and 37°C and resulting hemolytic phenotypes.

		Phenotype			
		Bovine blood		Sheep blood	
Isolate	Species	15°C	37°C	15°C	37°C
12396_bac	*Bacillus* sp.	γ	α, β−	γ	α, β−
12403-Bac	*Bacillus* sp.	γ	α, β−	γ	α, β−
L-1896	*Bacillus* sp.	γ	γ	γ	γ
L-1899	*Cryobacterium psychrotolerans*	γ	NG	γ	NG
L-1900	*Sphingomonas* sp.	γ	NG	γ	NG
L-1906	*Bacillus* sp.	γ	γ	γ	γ
L-1909	*Arthrobacter* sp.	γ	NG	γ	NG
L-1910	*Bacillus* sp.	γ	α	γ	α
L-1922	*Micrococcus* sp.	γ	β	γ	β
L-1964	*Pseudomonas* sp.	β+	NG	β+	NG
L-1969	*Pedobacter* sp.	β	β	β	β
L-1972	*Sphingomonas* sp.	γ	γ	γ	γ
L-1973	*Pedobacter* sp.	γ	β	γ	γ
L-1980	*Raoultella* sp.	γ	β	γ	β
L-1983	*Pseudomonas* sp.	γ	γ	γ	γ
L-1994	*Flavobacterium* sp.	β	γ	β	γ
L-1995	*Janthinobacterium* sp.	β	β	γ	β
L-2062	*Cryobacterium* sp.	γ	γ	γ	γ
L-2063	*Undibacterium* sp.	γ	NG	γ	γ
L-2137	*Pseudomonas* sp.	β−	β−	γ	γ
L-2142	*Pseudomonas* sp.	γ	β−	γ	γ
L-2145	*Pseudomonas* sp.	γ	β−	γ	γ
L-2265	*Pseudomonas* sp.	γ	β−	γ	γ
L-2266	*Janthinobacterium* sp.	γ	β−	γ	γ
L-2267	*Undibacterium* sp.	NG	NG	NG	NG
L-2270	Unidentified Oxalobacteraceae	NG	NG	NG	NG
L-2271	*Massilia* sp.	NG	NG	NG	NG
L-2273	*Sphingomonas* sp.	NG	NG	NG	NG
L-2275	*Massilia* sp.	NG	NG	NG	NG
L-2276	*Massilia* sp.	NG	NG	NG	NG
L-2279	*Cryobacterium* sp.	γ	α	γ	NG
L-2285	*Cryobacterium* sp.	β−	β−	γ	γ
L-2290	*Herminiimonas* sp.	NG	NG	NG	NG
L-2291	*Flavobacterium* sp.	NG	NG	NG	NG
L-2430	*Frigoribacterium* sp.	γ	β	γ	β
L-2433	*Curtobacterium* sp.	β+	NG	β	NG
L-2552	*Sphingomonas* sp.	γ	NG	γ	NG
L-2553	*Pseudomonas graminis*	γ	NG	γ	NG
L-2554	*Pseudomonas* sp.	γ	NG	γ	NG
L-2558	*Polaromonas* sp.	γ	NG	γ	NG
L-2560	*Flavobacterium* sp.	γ	NG	γ	NG
L-2571	*Cryobacterium* sp.	β−	β	γ	β
L-2573	*Flavobacterium* sp.	NG	NG	NG	NG
L-2575	*Pseudomonas* sp.	γ	NG	γ	NG
L-2577	*Massilia* sp.	NG	NG	NG	NG
L-2578	*Pseudomonas* sp.	γ	γ	γ	γ
L-2580	*Cryobacterium* sp.	γ	β	γ	β
L-2643	*Pseudomonas* sp.	β−	γ	γ	γ
L-2644	*Pseudomonas fluorescens*	β+	β−	β+	β−
L-2646	*Pseudomonas graminis*	γ	γ	γ	γ
L-2649	*Sphingomonas* sp. (*glacialis*)	γ	NG	γ	NG
L-2650	*Pseudomonas* sp.	β+	NG	β+	NG
L-2652	*Pseudomonas frederiksbergensis*	γ	γ	γ	β
L-2653	*Massilia* sp.	γ	NG	γ	NG
L-2656	*Pseudomonas fluorescens*	β+	β	β	γ
L-2657	*Pseudomonas* sp.	β+	NG	β	β
L-2658	*Pseudomonas fluorescens*	β+	NG	β	NG
L-2659	*Pseudomonas* sp.	β+	NG	β+	NG
L-2694	*Sphingomonas* sp.	γ	β	γ	α−
L-2696	*Pseudomonas* sp.	β−	γ	γ	β
An34	*Leifsonia* sp.	γ	α−	γ	α−
An58	*Enterococcus* sp.	γ	α−	α	γ
S3	*Micromonospora* sp.	γ	β	γ	β
S7	*Bacillus* sp.	β	β	β	β
S8	*Microbacterium* sp.	γ	α−	γ	α−
S10	*Oerskovia* sp.	α	α	α	β
S23b	*Microbacterium* sp.	γ	α	γ	β
S24	*Brevibacterium* sp.	γ	β−	γ	α
S26	*Microbacterium* sp.	γ	α	α	α
S27b	*Carnobacterium* sp.	α	α−	α	γ
S32	*Tessaracoccus* sp.	γ	α	α−	α
S44	*Bacillus* sp.	γ	β+	γ	β+
S58	*Salinibacterium* sp.	γ	α−	γ	α
S60	*Microbacterium* sp.	γ	α−	γ	α
S70	*Bacillus* sp.	γ	α	γ	α
S71	*Bacillus* sp.	γ	α−	γ	β
N2	*Exiguobacterium* sp.	γ	α−	γ	α−
N7	*Paenibacillus* sp.	γ	γ	γ	γ
N18	*Micromonospora* sp.	β	β+	β	β
N23	*Bacillus* sp.	γ	β+	γ	β+
N24	*Bacillus* sp.	γ	β+	γ	β+
N28	*Streptomyces* sp.	β−	γ	β+	β
N34	*Bacillus* sp.	β	β+	β−	β
N36a	*Pedobacter* sp.	α	α	β+	α
N39	*Carnobacterium* sp.	α	β	α	γ
N40	*Pseudomonas* sp.	β+	NG	β	NG
N41	*Streptomyces* sp.	γ	NG	γ	NG
N42	*Streptomyces* sp.	α	β	γ	β
N54	*Psychrobacillus* sp.	γ	γ	γ	γ
N58	*Carnobacterium* sp.	α+	NG	α+	NG
N61	*Psychrobacter* sp.	γ	NG	γ	NG
N71	*Pseudomonas* sp.	β+	NG	β+	NG
N83	*Bacillus* sp.	γ	β	γ	β
N106	*Salinibacterium* sp.	γ	α−	γ	α−

*Hemolytic phenotypes: α, α-hemolytic; β, β-hemolytic; γ, non-hemolytic; +, signifies a stronger phenotype; −, signifies a weaker phenotype; NG, no growth.*

*Bacillus* spp. exhibited greater temperature range tolerance, with all isolates growing at both 15 and 37°C. Although β-hemolytic phenotypes were common at 37°C (11/13 isolates), at the lower temperature the phenotypes were mostly non-hemolytic. Two isolates, S7 and N34, expressed β-hemolysis at both temperatures and on both blood types. Of the *Micromonospora* spp., strain N18 presented β-hemolytic activity at both temperatures and blood types, while strain S3 was β-hemolytic only at 37°C in both blood types. Isolates of *Carnobacterium* spp. displayed the strongest α hemolysis of all the tested bacteria (Figure 2B). Interestingly, isolates L-1994 (*Flavobacterium* sp.) and L-1995, (*Janthinobacterium* sp.), obtained from tap water from Svalbard, that also showed β-hemolytic activity and the only isolate belonging to the Enterobacteriaceae family (L-1980, *Raoultella* sp.) displayed a β-hemolytic activity at 37°C, whereas they were non-hemolytic at 15°C.

In general, isolates that were non-hemolytic on bovine blood plates were also non-hemolytic on sheep blood plates (Table 2) except for *Pseudomonas* spp. L-2652 (β-hemolytic only on sheep blood at 37°C) and L-2696 (β-hemolytic at 15°C on bovine blood and at 37°C on sheep blood), *Enterococcus* sp. An58 (α-hemolytic at 37°C on bovine and at 15°C on sheep blood), and *Pedobacter* sp. N36a (β-hemolytic at 15°C on sheep blood but α-hemolytic at 37°C and at both temperatures on bovine blood). About 30% of the isolates showed non-hemolytic phenotypes on both blood types, e.g., *Sphingomonas* sp., *Psychrobacillus* sp. Species belonging to the genera *Herminiimonas* sp. and *Massilia* sp. did not grow at any temperature or blood type used in this experiment. The three control strains displayed their corresponding hemolytic phenotype on blood agar plates.

### Hemolytic Assay With Erythrocyte Suspension

The second experiment involved the observation of hemolytic activity of the ethanolic extracts of bacterial lysates in a short assay (within a 60-min time frame) using erythrocyte suspensions. The objective of this experiment was to determine the potential presence of membrane-active secondary metabolites which could be present in the tested bacteria, and which could contribute to the pathogenicity of the producing organism.

**FIGURE 3:**
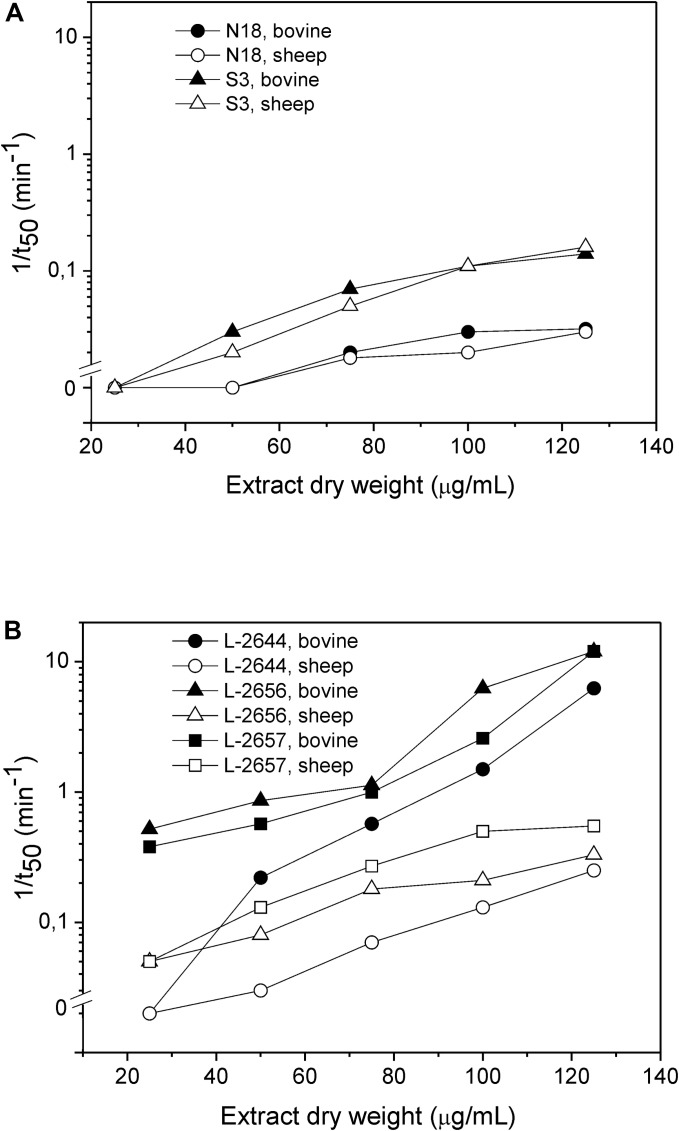
Hemolytic activity of ethanolic extracts of *Micromonospora* sp. **(A)** and *Pseudomonas* sp. **(B)**. Dependence of the rate of hemolysis, 1/*t*_50_, on the dry extract concentration. The hemolytic activity was assayed against bovine (full symbols) and sheep (open symbols) erythrocytes.

In the assays with bovine and sheep erythrocytes in suspension, only 5 extracts in total were hemolytic within 60 min of the assay: *Pseudomonas* sp. L-2644, L-2656, and L-2657 together with *Micromonospora* sp. S3 and N18 (Figure 3 and Supplementary Figure S1). *Pseudomonas* spp. showed faster hemolytic reactions on both blood types, compared to *Micromonospora* sp. In general, for the *Pseudomonas* sp. extracts higher values of 1/*t*_50_ were observed in bovine blood compared to sheep blood (Figure 3). Ethanolic extracts of the three control species displaying the hemolytic phenotype on blood agar plates were inactive on erythrocytes in suspension.

### Antimicrobial Susceptibility Test

In total, 118 isolates were tested for antimicrobial resistance. The plates were read as soon as colonies were detected on them and the results are presented in Table 3 (summary) and Supplementary Table S3 (all results), except for 14 isolates that did not grow on the used media or in the presence of any of the antimicrobials tested. TET was the most effective antimicrobial, with most isolates (95/104) being susceptible, followed by KAN (84/104 susceptible isolates). At the other end of the spectrum, IPM was the least effective antimicrobial at the used concentration, with only 21/104 susceptible isolates. Resistant strains to higher concentrations of imipenem are mostly from the genera *Flavobacterium*, *Enterococcus*, *Janthinobacterium*, *Raoultella*, and, notably, *Pseudomonas*. Interestingly, not all *Pseudomonas* spp. behave alike. For instance, strains L-2652 (closely related to *P. frederiksbergensis*) and L-2553 and L-2646 (both closely related to *P. graminis*) were not resistant to >4 m/L of imipenem in the media. The following IPM-resistant strains were also carbapenemase producers (OXA-48 and other carbapenemases such as KPC, MBL, or NDM): *Flavobacterium* sp. (L-1994), *Janthinobacterium* sp. L-1995, *Pseudomonas* sp. N71 (L-5704), L-1983, L-2137, L-2142, L-2145, L-2554, L-2265, L-2575, L-2578, L2643, L-2644, L2650, L-2656, L-2658, and *Raoultella* sp. L-1980 (Supplementary Table S4).

**TABLE 3:** Summary of results for antimicrobial susceptibility for the 104 isolates tested, showing number of isolates that are: − susceptible; (−) moderately susceptible; (+) moderately resistant; + resistant; M indicates the appearance of individual mutant colonies.

Summary	Growth	AMP	CHL	CIP	CTX	ERY	IPM	KAN	TET
−	17	74	77	47	41	64	21	84	95
(−)	9	2	4	11	9	6	13	9	7
(+)	11	1	1	5	5	7	9	4	0
+	67	27	22	17	49	27	61	6	2
M	0	0	0	24	0	0	0	1	0

After a week of incubation, resistant colonies (possible mutants) appeared only on plates containing CIP (24/104) and KAN (1/104). About 50% of the isolates (54/104) were resistant to CTX.

The species that were resistant to TET belonged to five genera only: *Pseudomonas* (L-1964, L-1983, L-2137, L-2142, and L-2643), *Janthinobacterium* (L-1995), *Oerskovia* (S10), *Flavobacterium* (L-1994), and *Rahnella* (L-2695). The isolates mentioned above (of which two were obtained from tap water), shared very similar antimicrobial resistance profiles, i.e., resistance to all antimicrobials tested except for KAN and individual mutant colonies for CIP.

All *Pseudomonas* sp. were resistant to multiple antimicrobials, including AMP, CHL, CTX, ERY, and IPM. However, the two isolates belonging to *Pseudomonas graminis* were susceptible to AMP, CHL, CIP, ERY, KAN, and TET. The majority (18/24) of individual mutant colonies were also observed among the *Pseudomonas* spp. isolates.

Generally, *Bacillus* spp. were susceptible to the majority of the antimicrobials tested (AMP, CHL, ERY, KAN, and TET), with a few exceptions in case of CIP, IPM, and CTX. *Micromonospora* spp. were predominantly susceptible to the antimicrobials tested, with resistance detected for CTX and ERY.

Isolates belonging to the Enterobacteriaceae family (i.e., *Rahnella* sp. L-2695 and *Raoultella* sp. L-1980) were resistant to AMP and IPM. Some isolates of the genera *Cryobacterium* (L-2062, L-2285, L-2571, and L-2580), *Massilia* (L-2271) and *Undibacterium* (L-2063 and L-2267), did not show growth on the control plates (nutrient agar without added antimicrobials) but generally showed better growth on the plates containing IPM.

## Discussion

Until recently, the Arctic represented a pristine environment, largely unaffected by anthropogenic influences. However, this geographic area is now experiencing the dramatic repercussions of climate change more than other regions on the planet (Intergovernmental Panel on Climate Change [IPCC], 2007). Due to its low adaptability, these consequences are amplified (Doney et al., 2012) and the stressful conditions they bring about might cause the release of environmental bacteria with potential virulence-associated phenotypes, e.g., compounds with hemolytic activity and antimicrobial resistance. Moreover, bacterial species in the short term are expected to come in contact more frequently with humans, animals, and plants outside of their current environments (Epstein, 2001; Cavicchioli et al., 2019; Hutchins et al., 2019).

### Majority of Psychrotolerant Bacterial Isolates Are Able to Grow at 37°C

In this study, more than 70% of the culturable species obtained from the Arctic environmental samples were psychrotolerant, with an optimal growth temperature of about 15°C. Although a growth temperature range of 8–20°C (Moyer and Morita, 2007) should define psychrotolerant microorganisms, the majority of our psychrotolerant strains were able to grow at 37°C, indicating a broader range of temperature adaptation (Tindall, 2004). Interestingly, the 13 isolates obtained from incubation at 15–17°C that were not able to grow at 37°C might be considered as true psychrotolerants or true psychrophiles with slower growth rates at higher temperature, and a broader temperature growth range (Tindall, 2004; Moyer and Morita, 2007).

### Environmental Strains Produce Compounds With Hemolytic Activity Expressed Differently at Low (15°C) and High (37°C) Temperatures

A considerable number of isolates in this study showed a hemolytic phenotype when cultured on blood agar plates, in accordance to recent reports (Mogrovejo-Arias et al., 2020). The type of blood used (bovine vs. sheep) did not affect hemolytic activity. Both α- and β-hemolytic phenotypes are considered as a virulence-associated determinant and of clinical relevance when assessing the significance of the hemolytic environmental species and/or strains (Bayley, 1997; Ramachandran, 2013).

While most of the bacterial isolates cultured on agar plates showed active growth, some genera (*Flavobacterium* sp., *Herminiimonas* sp., *Massilia* sp., *Sphingomonas* sp., and *Undibacterium* sp.) could not be assessed due to lack of growth, which was possibly inhibited by the high nutrient content of the culture medium used for this test (Tindall, 2004). In addition to an incubation temperature of 15°C, a suitable temperature for the growth of psychrotolerant bacteria, the blood agar plates were also incubated at 37°C in order to assess the hemolysis at a clinically relevant temperature and to investigate the influence of the temperature on the hemolysis activity. Several genera responded differently to the incubation temperature. For instance, *Pseudomonas* spp. had mostly hemolytic phenotypes at 15°C while *Bacillus* spp. expressed hemolytic activity mainly at 37°C. Differences in the hemolysins structures and modes of action might explain that some of them have a lytic effect only above certain temperatures, at which the characteristics of erythrocyte membranes change due to increased membrane fluidity (Carr et al., 2001; Asam et al., 2015). Alternatively, the expression of hemolytic genes might be temperature-regulated (as described by Madrid et al., 2002) and could account for the differences observed in our experiments.

### *Pseudomonas* sp. Organic Extracts Show Faster Hemolytic Response in Bovine Erythrocytes Suspension Than in Sheep Erythrocytes Within 60 min

Our study comprises a large and preliminary screening study of crude extracts where non-purified membrane-active compounds were present. To the best of our knowledge, it represents the first screening of this sort for hemolytic activity in Arctic environmental bacteria. The hemolytic activity of ethanolic extracts was tested in two types of erythrocyte suspensions: sheep and bovine. Sheep blood is commonly used as a standard for the determination of the hemolytic phenotype (Yeh et al., 2009; Atlas, 2010) and bovine erythrocytes have been previously used to determine the hemolytic capabilities of environmental extracts from different habitats and organisms (Sepčić et al., 2011).

The hemolytic potential of human-associated, pathogenic or opportunistic bacteria is regularly assessed using animal blood (Bevivino et al., 2002; González-Rodríguez et al., 2007) as it is suitable for carrying out microbiological tests used in routine identification and susceptibility profiling of known human pathogens (Yeh et al., 2009).

Anuclear, mature red blood cells are often used as a model to assess the membrane damage, either by monitoring the hemoglobin release or by measuring the turbidity of erythrocyte suspension. However, it should not be inferred that erythrocytes are the only target cells of the compounds observed in this study (Tomita and Kamio, 1997) given that hemolytic compounds can often also be cytolytic for cells of the immune system, e.g., macrophages and neutrophils (Asam et al., 2015), which, in the case of pathogenic organisms, greatly increases their potential virulence (Billington et al., 2000).

The discrepancies in membrane composition among different mammalian erythrocytes influence the hemolytic activity of hemolysins, which act by recognizing specific membrane lipids or membrane receptors (DuMont and Torres, 2014; Rojko and Anderluh, 2015). We observed that the lytic activity of the tested *Pseudomonas* sp. extracts was more pronounced on bovine erythrocytes than on the sheep ones (Figure 3). Red blood cell membranes from different mammals display different composition and physical characteristics (e.g., fluidity). Sheep erythrocytes contain a higher percentage of choline phospholipids (with sphingomyelin representing more than 50% of all the phospholipids) and acidic phospholipids, but a lower content of phosphatidylethanolamine compared to bovine erythrocytes (Bernheimer, 1974; Virtanen et al., 1998; Windberger et al., 2003). Cholesterol content, on the other hand, is higher in bovine red blood cells than in sheep ones (Nelson, 1967). Thus, the faster hemolytic reaction on bovine erythrocytes observed might be because the hemolytic compound(s) produced by the isolates in this study have greater affinity for cholesterol-enriched membrane domains.

Based on our results, the use of more than one type of blood is recommended as it widens the spectrum of observed hemolytic activities. Future research could benefit from the inclusion of assays with human blood, the results of which could be predicted to some extend based on similarities with the types of blood used in the present study.

### Blood Agar and Turbidimetric Assays Highlight Different Compounds Responsible for Hemolysis

A greater number of isolates were found to have hemolytic activity on blood agar plates (around 30 isolates) vs. the activity of their ethanolic extracts on erythrocyte suspensions (5 isolates).

The culture conditions at which the isolates were grown in preparation for each test, i.e., solid media in agar plates vs. liquid media used for the suspension test, could account for the difference in the number of positive phenotypes as they directly affect gene expression (Favero et al., 2014).

Another possible explanation for such a remarkable difference between the tests is that each one assesses the presence of different compounds. Plates show the effects of a wider range of molecules produced by the bacteria in the course of their growth (e.g., proteinaceous endotoxins, exotoxins, pigments, and secondary metabolites). On the other hand, for the suspensions test, a limited selection of compounds was obtained in the extraction solvent (96% ethanol in this case). Ethanol allows the extraction of small, less polar molecules or secondary metabolites and likely excludes proteins (Sepčić et al., 2011). Crude extracts, moreover, might possess more than one hemolytically active and less polar compound, mainly acting as surfactants and whose function is independent of membrane lipid composition. This might explain the observed absence of hemolytic activity of the ethanolic extract of *S. pyogenes*, a bacterium commonly used as a positive β-hemolytic control in blood agar tests. In fact, the main hemolytic compounds characteristic of *S. pyogenes* are pore-forming proteinaceous cytotoxins streptolysin O and streptolysin S (Hynes and Sloan, 2016), which cannot be extracted with ethanol.

Finally, the growth phase of the culture at the moment of the test might vary, directly affecting the compounds produced by the bacteria. That is, compounds produced in actively growing cultures (for instance, liquid cultures) differ from those produced in cultures going through stationary phase (for instance, solid cultures).

### Arctic Environmental Strains Show Widespread Resistance to Commercial Antimicrobials

Studying antimicrobial resistance profiles in Arctic isolates is gaining in importance since climate change is exerting a stronger pressure on this environment and higher temperatures are associated with increased antimicrobial resistance in common pathogens (Hutchins et al., 2019).

No breakpoint tables for Minimum Inhibitory Concentration or Zone Diameters have been established for Arctic species/strains (European Committee on Antimicrobial Susceptibility Testing [EUCAST], 2019). Therefore, the definition of resistance for these bacteria is difficult to ascertain. In this study, we performed screening for phenotypic resistance on media supplemented with antimicrobials as an indicator for the presence of resistance genes.

On one hand, we observed that a majority of the Arctic environmental isolates analyzed were resistant to at least one of the antimicrobials tested, suggesting a strong competitiveness in the habitat. Some isolates were resistant even toward broad-spectrum antimicrobials, such as ciprofloxacin and chloramphenicol. Similar results were found in other natural environments such as Antarctica (Tam et al., 2015), high-altitude wetlands in Argentina (Dib et al., 2008), and Siberia (Mindlin et al., 2008). On the other hand, almost all of the isolates were susceptible to tetracycline, contrary to what has been reported before for other cold environments (Perron et al., 2015). Since tetracycline could be naturally occurring in soils, such widespread susceptibility is surprising, but could be due to a lack of tetracycline producers in the Arctic. In accordance to our findings, Pal et al. (2016) reported that tetracycline resistance dominates human, human-impacted and animal microbiomes but not so far natural environments. The mutant phenotypes observed for ciprofloxacin are usually induced by low concentrations of this antimicrobial (Cirz and Romesberg, 2006).

Resistance to some antibiotics can occur quickly during selective enrichment, predominantly that which is triggered by point mutations in housekeeping genes, e.g., ribosomal proteins and efflux pumps. Accordingly, growth observed in plates containing ciprofloxacin, erythromycin, and kanamycin has been cautiously interpreted. The constant appearance of a low number of individual, presumably mutant, colonies is indicated in Supplementary Table S3.

Isolates belonging to the genus *Pseudomonas* were the ones with the strongest multidrug-resistant abilities, showing marked resistance tetracycline, and with frequent occurrence of individual mutant ciprofloxacin resistant colonies. Correspondingly, *Pseudomonas* spp. isolated from Antarctica showed a variety of antimicrobial resistant profiles (Tam et al., 2015). *Pseudomonas* sp. are known for its extended resistances to antimicrobials (European Committee on Antimicrobial Susceptibility Testing [EUCAST], 2019) and are known to possess capsules that might aid in lowering the concentration of antimicrobial that reaches the cells. In addition, *Pseudomonas* sp. possess efflux pumps which contribute to the multidrug resistance phenotype and might explain the mutant phenotypes observed (Okamoto et al., 2002; Aeschlimann, 2003). Even though efflux pumps can be also associated with other functions in the cell, the responsible genes can be transferred to other environments and into other species where the resistance phenotype becomes of clinical relevance (Graham et al., 2019).

Resistance to cefotaxime was observed in 50% of our isolates. Resistant phenotypes against this extended-spectrum β-lactam is considered of clinical relevance (Mir et al., 2016), especially among Gram-negative species and notoriously resistant *Pseudomonas* sp.

Finally, we observed that the concentration of imipenem used in this study favored, rather than inhibited, the growth of several isolates, notably *Massilia* sp. and *Cryobacterium* sp. As far as we can tell from searching the available literature, such observation hasn’t been reported yet. Phenotypic resistance to imipenem is of high importance since carbapenemase-producing bacteria have become a major public health concern worldwide. In this study, we identified carbapenem resistant bacteria and predicted carbapenem resistance mechanisms (CRM) and the subsequent potential for horizontal spread. While the best known and currently most important CRM have been described in members of the Enterobacteriaceae family and for *Pseudomonas aeruginosa*, literature data about CRM in Gram-positive bacteria is scarce.

We have observed a variety of resistotypes for carbapenem resistance (CR) among several, but not all, *Bacillus* sp. isolates. Further, we retrieved CR isolates from the genera *Cryobacterium*, *Leifsonia*, and *Streptomyces*. This observation, in accordance with CR in Gram-positive bacteria, is likely the result of substitutions in amino acid sequences of penicillin-binding proteins (PBPs) or acquisition/production of new carbapenem-resistant PBPs (Papp-Wallace et al., 2011). Although chromosomally encoded, these alleles could spread horizontally by transformation or transduction.

Acquired carbapenem resistance in Gram-negative bacteria is a consequence of enzymatic inactivation of the drug, target site mutation and efflux pumps (Codjoe and Donkor, 2018). We hypothesize on putative resistance mechanisms on the basis of the most commonly described mechanism for strains of the same species/genus or other closely related bacteria, and our results of differential growth on nutrient agar plates supplemented with different concentrations of imipenem and commercial CHROMID ^®^ CARBA SMART agar plates. The majority of carbapenem resistant *Pseudomonas* isolates grew in plates containing 4–10 mg/L of imipenem and on both sections of the CARBA SMART plates. This indicates the presence of putative carbapenemase enzymes. *Pseudomonas* sp. L-2696, grew on nutrient agar plates with 10 mg/L of imipenem but not on CARBA SMART plates, indicating putative efflux pumps and/or target site mutation. Further studies, including cloning, would clarify the possibility and subsequent threat of horizontal transfer to clinically important *Pseudomonas* strains. *Flavobacterium* sp. L-1994 grew on both CARBA SMART sections, thus possibly encoding a carbapenemase. Naas et al. (2003) described the JOHN-1 β- metallo-β-lactamase from an *Flavobacterium johnsoniae*, an environmental plant pathogen which can also cause skin lesions in fish (Naas et al., 2003) and Kuai et al. (2011) described a KPC-2 enzyme from a *Flavobacterium odoratum* strain (Kuai et al., 2011). Carbapenemase genes have also been described from other members of the Flavobacteriaceae family, such as the chromosome-encoded metallo-β-lactamases MUS-1 and MUS-2 in *Myroides* and carbapenemases from *Chryseobacterium* strains (Al-Bayssari et al., 2015; Gudeta et al., 2016). *Janthinobacterium* sp. L-1995 grew on nutrient agar plates with imipenem at concentrations up to 4 mg/L and on both media on CARBA SMART plates. Carbapenem resistance in member of this genus has been described by Rossolini et al. (2001) and also observed by Gudeta et al. (2016). The described carbapenemases were chromosomally encoded and not proven yet to be horizontally transferable. The most interesting isolates, given the relatedness to carbapenem resistant Enterobacteriaceae are *Rahnella* sp. L-2695 and *Raoultella ornithinolytica* L-1980. The former was negative for carbapenemase and positive for OXA-48-like hydrolytic activity and the later was positive for both. Besides the fact, that the presence of carbapenemases genes has been confirmed several times for *Raoultella* spp. a large plasmid carrying genes for NDM-1- and CTX-M-3 β-lactamases, has been detected in a carbapenemase-producing *R. ornithinolytica* strain from stool samples recently (Wang et al., 2019). Particularly interesting is the *Rahnella* sp. isolate L-2695, since it is, in addition to showing OXA-48-like carbapenemase activity, one of only two tetracycline resistant isolates. Integrons and chromosomal extended-spectrum class A β-lactamase have already been described in members of this genus (Ruimy et al., 2010; Koczura et al., 2016). To summarize; we predict chromosome encoded β-lactamases for the majority of the isolated imipenem resistant strains. According to literature data, the presence of plasmid or phage encoded resistance genes is less likely. Nevertheless, horizontal transfer events of resistance genes is a possible event, predominantly through transduction by phages.

## Conclusion

To the best of our knowledge, this study is among the first screenings of Arctic environmental bacteria for hemolytic activity, a common bacterial virulence-associated phenotype, in extreme habitats. Hemolytic activity was temperature-dependent and observed in a third of the tested isolates on blood agar plates with both sheep and bovine blood types. In erythrocyte suspensions, the hemolysis occurred only in five isolates belonging to genera *Pseudomonas* and *Micromonospora*. The type of erythrocytes appears to influence the reaction time for the hemolytic compounds, as bovine blood was more readily lysed than sheep blood with *Pseudomonas* sp. extracts. In addition, the Arctic strains tested showed resistance to several commonly used and clinically relevant antimicrobials. However, the number of kanamycin and tetracycline resistant strains was rather low. Imipenem was the antimicrobial with most resistant isolates, possibly due to the low concentration tested or the widespread presence of chromosome encoded β-lactamases. In the context of our study, *Pseudomonas* was the genus with the highest potential clinical relevance: hemolytic on blood agar plates, hemolytic on erythrocyte suspensions and with a broad resistance profile to the antimicrobials tested. Studying potential pathogenic phenotypes of environmental strains provides insights into possible evolutionary adaptations and origins of clinically relevant bacteria and helps assessing the possible threats Arctic bacteria represent in other environments.

## Data Availability Statement

The datasets generated for this study can be found in GenBank with the following accession numbers: MK453054–MK453127, MK670504–MK670553, MN161207–MN161227, MH714605–MH714685, and MN450679–MN450730.

## Author Contributions

DM and LP collected the samples, performed the wet lab analyses, interpreted the data, and wrote the manuscript with input from all co-authors. CG, MT, JA-A, and KS designed and guided the execution of all experiments. MT and JA-A performed additional experiments with the imipenem-resistant strains. FB and NG-C supervised the project and contributed to the interpretation of the results and valuable discussion.

## Conflict of Interest

DM was employed by Dr. Brill + Partner GmbH as part of a research network (European Union’s Horizon 2020 Research and Innovation Programme, Marie Skłodowska-Curie grant agreement no. 675546). FB is Managing Director of Dr. Brill + Partner GmbH. The remaining authors declare that the research was conducted in the absence of any commercial or financial relationships that could be construed as a potential conflict of interest.

## Footnotes

1 https://blast.ncbi.nlm.nih.gov/Blast.cgi
