# Supplementary material for: Prevalence of Antimicrobial Resistance and Hemolytic Phenotypes in Culturable Arctic Bacteria
Source: Front Microbiol. 2020 Apr 3;11:570. doi: 10.3389/fmicb.2020.00570 (PMC7147505; doi:10.3389/fmicb.2020.00570)
Supplement: Supplementary file 1 [file Data_Sheet_1.docx]

# **SUPPLEMENTARY MATERIAL**

**Article title**

**Authors:** D.C. Mogrovejo^1+^, L. Perini^2+^**^*^**, C. Gostinčar^2,3^, K. Sepčić^2^, M. Turk^2^, J. Ambrožič-Avguštin^2^, F. H. H. Brill^1^, N. Gunde-Cimerman^2^

^+^ Equally contributing first authors.

*Corresponding author:

**TABLES:**

Table S1. All culturable bacterial isolates obtained during this study

Table S2. Isolates used for hemolytic activity and antimicrobial susceptibility testing

Table S3. List of the isolates selected for the antimicrobial susceptibility testing with the corresponding AMR profile

Table S4. List of the isolates with imipenem resistance further inoculated on nutrient agar plates containing 4, 6, 8 and 10mg/l of imipenem and on CHROMID® CARBA SMART Agar, for the detection of carbapenemases.

**FIGURES:**

Figure S1. Phylogenetic analyses of the 16S sequences of *Pseudomonas* species (A) and *Micromonospora* species (B) from Arctic environments compared with sequences of type strains or other closest species deposited in the GenBank database. The tree was constructed using the maximum composite likelihood method. Bootstrap values are shown (500 replicates). The sequence of *Pseudomonas aeruginosa* (NR_114471) (A) and *Micromonospora vinacea* (NR_151946) (B) were used as outgroups, respectively. Evolutionary analyses were conducted in MEGA

**Supplementary table S1. All culturable isolates obtained for this study**

| Phylum | Isolate (EXB) | Species | Gram stain | Id. % closest neighbor | Sample | Sampling location | Sampling year | Isolation temp. (°C) | Filtrated volume (ml) | Genbank accession number |
| --- | --- | --- | --- | --- | --- | --- | --- | --- | --- | --- |
| Actinobacteria | An32 | *Agreia* sp. | Positive | 99 | Snow | Svalbard - Longyearbyen | 2016 | 5 | 50 | MN450680 |
|  | S28 | *Agreia* sp. | Positive | 99 | Snow | Svalbard - Longyearbyen | 2016 | 17 | 50 | MH714665 |
|  | S29 | *Agreia* sp. | Positive | 99 | Snow | Svalbard - Longyearbyen | 2016 | 17 | 50 | MN450686 |
|  | S50 | *Agreia* sp. | Positive | 99 | Snow | Svalbard - Longyearbyen | 2016 | 17 | 50 | MN450691 |
|  | S54 | *Agrococcus* sp. | Positive | 99 | Sea water | Svalbard - Longyearbyen | 2016 | 5 | 50 | MH714671 |
|  | L-1911 | *Arthrobacter* sp. | Positive | 99 | Cryoconite | Greenland Ice Sheet | 2016 | 15 | 0.1 | MK453113 |
|  | L-1909 | *Arthrobacter* sp. | Positive | 100 | Dispersed cryoconite | Greenland Ice Sheet | 2016 | 15 | 0.1 | MK453111 |
|  | An49 | *Arthrobacter* sp. | Positive | 98 | Sediment | Svalbard - Longyearbyen | 2016 | 5 | n/a | MN450683 |
|  | N62 | *Arthrobacter* sp. | Positive | 99 | Sediment | Svalbard - Ny Alesund | 2017 | 17 | n/a | MN450724 |
|  | N66 | *Arthrobacter* sp. | Positive | 99 | Soil | Svalbard - Ny Alesund | 2017 | 37 | n/a | MN450725 |
|  | N79 | *Arthrobacter* sp. | Positive | 99 | Soil | Svalbard - Ny Alesund | 2017 | 37 | n/a | MN450727 |
|  | N84 | *Arthrobacter* sp. | Positive | 99 | Melt water | Svalbard - Ny Alesund | 2017 | 17 | 2000 | MH714643 |
|  | S16 | *Arthrobacter* sp. | Positive | 99 | Sediment | Svalbard - Longyearbyen | 2016 | 17 | n/a | MH714657 |
|  | S25 | *Arthrobacter* sp. | Positive | 98 | Sediment | Svalbard - Longyearbyen | 2016 | 17 | n/a | MH714662 |
|  | S34 | *Arthrobacter* sp. | Positive | 99 | Snow | Svalbard - Longyearbyen | 2016 | 5 | 5 | MH714668 |
|  | S61 | *Arthrobacter* sp. | Positive | 99 | Sea water | Svalbard - Longyearbyen | 2016 | 17 | 50 | MN450693 |
|  | S68 | *Arthrobacter* sp. | Positive | 99 | Sediment | Svalbard - Longyearbyen | 2016 | 17 | n/a | MN450695 |
|  | S81 | *Arthrobacter* sp. | Positive | 98 | Sediment | Svalbard - Longyearbyen | 2016 | 17 | n/a | MH714681 |
|  | S31 | *Blastococcus* sp. | Positive | 99 | Sea water | Svalbard - Longyearbyen | 2016 | 17 | 50 | MH714666 |
|  | S24 | *Brevibacterium* sp. | Positive | 99 | Sediment | Svalbard - Longyearbyen | 2016 | 17 | n/a | MH714661 |
|  | L-1898 | *Cryobacterium psychrotolerans* | Positive | 100 | Cryoconite | Greenland Ice Sheet | 2016 | 15 | 0.1 | MK453103 |
|  | L-1899 | *Cryobacterium psychrotolerans* | Positive | 100 | Cryoconite | Greenland Ice Sheet | 2016 | 15 | 0.1 | MK453104 |
|  | L-2061 | *Cryobacterium psychrotolerans* | Positive | 100 | Dark ice | Greenland Ice Sheet | 2016 | 15 | 0.1 | - |
|  | An24 | *Cryobacterium* sp. | Positive | 99 | Sediment | Svalbard - Longyearbyen | 2016 | 17 | n/a | MH714606 |
|  | An33 | *Cryobacterium* sp. | Positive | 99 | Sea water | Svalbard - Longyearbyen | 2016 | 5 | 50 | MN450681 |
|  | L-2263 | *Cryobacterium* sp. | Positive | 100 | Clear ice | Greenland Ice Sheet | 2017 | 15 | 100 | MK453057 |
|  | L-2431 | *Cryobacterium* sp. | Positive | 99 | Clear ice | Greenland Ice Sheet | 2017 | 15 | 100 | MK453056 |
|  | L-2136 | *Cryobacterium* sp. | Positive | 99 | Clear ice | Greenland Ice Sheet | 2017 | 15 | 100 | MK453058 |
|  | L-2062 | *Cryobacterium* sp. | Positive | 100 | Dark ice | Greenland Ice Sheet | 2016 | 15 | 0.1 | MN161217 |
|  | L-2279 | *Cryobacterium* sp. | Positive | 97 | Subglacial ice | Svalbard - Ny Alesund | 2017 | 15 | 5 | - |
|  | L-2285 | *Cryobacterium* sp. | Positive | 99 | Subglacial ice | Svalbard - Ny Alesund | 2017 | 15 | 5 | MK670539 |
|  | L-2568 | *Cryobacterium* sp. | Positive | 99 | Subglacial ice | Svalbard - Ny Alesund | 2017 | 15 | 5 | MK670530 |
|  | L-2571 | *Cryobacterium* sp. | Positive | 100 | Subglacial ice | Svalbard - Ny Alesund | 2017 | 15 | 5 | MK670534 |
|  | L-2579 | *Cryobacterium* sp. | Positive | 99 | Subglacial ice | Svalbard - Ny Alesund | 2017 | 15 | 5 | MK670543 |
|  | L-2580 | *Cryobacterium* sp. | Positive | 99 | Subglacial ice | Svalbard - Ny Alesund | 2017 | 15 | 5 | MK670544 |
|  | N91 | *Cryobacterium* sp. | Positive | 100 | Glacial meltwater | Svalbard - Ny Alesund | 2017 | 17 | 1000 | MH714646 |
|  | S55 | *Cryobacterium* sp. | Positive | 99 | Sea water | Svalbard - Longyearbyen | 2016 | 5 | 50 | MN450692 |
|  | L-2433 | *Curtobacterium* sp. | Positive | 100 | Dark ice | Greenland Ice Sheet | 2017 | 15 | 0.1 | MK453060 |
|  | L-2264 | *Curtobacterium* sp. | Positive | 100 | Snow | Greenland Ice Sheet | 2017 | 15 | 100 | MK453059 |
|  | L-2550 | *Frigoribacterium* sp. | Positive | 99 | Clear ice | Greenland Ice Sheet | 2017 | 15 | 100 | MK453061 |
|  | L-2548 | *Frigoribacterium* sp. | Positive | 99 | Dark ice | Greenland Ice Sheet | 2017 | 15 | 0.1 | MK453062 |
|  | L-2549 | *Frigoribacterium* sp. | Positive | 99 | Dark ice | Greenland Ice Sheet | 2017 | 15 | 0.1 | MK453063 |
|  | L-2551 | *Frigoribacterium* sp. | Positive | 99 | Dark ice | Greenland Ice Sheet | 2017 | 15 | 0.1 | - |
|  | L-2429 | *Frigoribacterium* sp. | Positive | 99 | Supraglacial water | Greenland Ice Sheet | 2017 | 15 | 0.1 | MK453064 |
|  | L-2430 | *Frigoribacterium* sp. | Positive | 99 | Supraglacial water | Greenland Ice Sheet | 2017 | 15 | 0.1 | MK453065 |
|  | N47 | *Frondihabitans* sp. | Positive | 99 | Soil | Svalbard - Ny Alesund | 2017 | 17 | n/a | MH714631 |
|  | S22 | *Gordonia* sp. | Positive | 99 | Sea water | Svalbard - Longyearbyen | 2016 | 17 | 50 | MH714659 |
|  | An34 | *Leifsonia* sp. | Positive | 98 | Sea water | Svalbard - Longyearbyen | 2016 | 5 | 50 | MH714607 |
|  | An36 | *Microbacterium* sp. | Positive | 99 | Sea water | Svalbard - Longyearbyen | 2016 | 5 | 50 | MN450682 |
|  | S23b | *Microbacterium* sp. | Positive | 98 | Sea water | Svalbard - Longyearbyen | 2016 | 37 | 50 | MH714660 |
|  | S26 | *Microbacterium* sp. | Positive | 98 | Sediment | Svalbard - Longyearbyen | 2016 | 17 | n/a | MH714663 |
|  | S60 | *Microbacterium* sp. | Positive | 98 | Sea water | Svalbard - Longyearbyen | 2016 | 5 | 50 | MH714673 |
|  | S75 | *Microbacterium* sp. | Positive | 99 | Sediment | Svalbard - Longyearbyen | 2016 | 17 | n/a | MN450697 |
|  | S8 | *Microbacterium* sp. | Positive | 99 | Sediment | Svalbard - Longyearbyen | 2016 | 37 | n/a | MH714654 |
|  | L-1922 | *Micrococcus* sp. | Positive | 99 | Clear ice | Greenland Ice Sheet | 2016 | 15 | 0.1 | MK453127 |
|  | N18 | *Micromonospora* sp. | Positive | 99 | Soil | Svalbard - Ny Alesund | 2017 | 37 | n/a | MH714618 |
|  | S3 | *Micromonospora* sp. | Positive | 99 | Sediment | Svalbard - Longyearbyen | 2016 | 37 | n/a | MH714651 |
|  | S10 | *Oerskovia* sp. | Positive | 99 | Sea water | Svalbard - Longyearbyen | 2016 | 37 | 50 | MH714655 |
|  | S11 | *Oerskovia* sp. | Positive | 100 | Sea water | Svalbard - Longyearbyen | 2016 | 37 | 50 | MN450685 |
|  | S73 | *Paeniglutamicibacter* sp. | Positive | 99 | Sediment | Svalbard - Longyearbyen | 2016 | 5 | n/a | MH714679 |
|  | An21 | *Propioniciclava* sp. | Positive | 93 | Sediment | Svalbard - Longyearbyen | 2016 | 17 | n/a | MH714605 |
|  | N106 | *Salinibacterium* sp. | Positive | 99 | Melt water | Svalbard - Ny Alesund | 2017 | 17 | 2000 | MH714650 |
|  | S58 | *Salinibacterium* sp. | Positive | 99 | Sea water | Svalbard - Longyearbyen | 2016 | 5 | 50 | MH714672 |
|  | S87 | *Sanguibacter* sp. | Positive | 99 | Sediment | Svalbard - Longyearbyen | 2016 | 17 | n/a | MH714683 |
|  | N28 | *Streptomyces* sp. | Positive | 99 | Soil | Svalbard - Ny Alesund | 2017 | 17 | n/a | MH714621 |
|  | N32 | *Streptomyces* sp. | Positive | 99 | Soil | Svalbard - Ny Alesund | 2017 | 17 | n/a | MN450713 |
|  | N41 | *Streptomyces* sp. | Positive | 99 | Soil | Svalbard - Ny Alesund | 2017 | 17 | n/a | MH714628 |
|  | N42 | *Streptomyces* sp. | Positive | 99 | Soil | Svalbard - Ny Alesund | 2017 | 17 | n/a | MH714629 |
|  | N51 | *Streptomyces* sp. | Positive | 99 | Soil | Svalbard - Ny Alesund | 2017 | 17 | n/a | MN450721 |
|  | N53 | *Streptomyces* sp. | Positive | 98 | Soil | Svalbard - Ny Alesund | 2017 | 37 | n/a | MN450722 |
|  | N70 | *Streptomyces* sp. | Positive | 99 | Soil | Svalbard - Ny Alesund | 2017 | 37 | n/a | MN450726 |
|  | N80 | *Streptomyces* sp. | Positive | 99 | Soil | Svalbard - Ny Alesund | 2017 | 37 | n/a | MN450728 |
|  | N99 | *Streptomyces* sp. | Positive | 99 | Soil | Svalbard - Ny Alesund | 2017 | 17 | n/a | MN450730 |
|  | S20 | *Subtercola* sp. | Positive | 99 | Snow | Svalbard - Longyearbyen | 2016 | 17 | 50 | MH714658 |
|  | S30 | *Subtercola* sp. | Positive | 99 | Snow | Svalbard - Longyearbyen | 2016 | 17 | 50 | MN450687 |
|  | S32 | *Tessaracoccus* sp. | Positive | 99 | Sea water | Svalbard - Longyearbyen | 2016 | 17 | 50 | MH714667 |
|  | S72 | *Tessaracoccus* sp. | Positive | 98 | Sediment | Svalbard - Longyearbyen | 2016 | 37 | n/a | MH714678 |
| Bacteroidetes | S65 | *Algoriphagus* sp. | Negative | 99 | Sediment | Svalbard - Longyearbyen | 2016 | 17 | n/a | MH714674 |
|  | L-1968 | *Dyadobacter* sp. | Negative | 98 | Lake ice | Svalbard - Longyearbyen | 2016 | 10 | 125 | MN161208 |
|  | L-2556 | *Flavobacterium sp.* | Negative | 99 | Glacial meltwater | Svalbard - Ny Alesund | 2017 | 15 | 10 | MK670507 |
|  | L-2559 | *Flavobacterium sp.* | Negative | 99 | Glacial meltwater | Svalbard - Ny Alesund | 2017 | 15 | 50 | MK670513 |
|  | L-1981 | *Flavobacterium sp.* | Negative | 99 | Sea water | Svalbard - Longyearbyen | 2016 | 10 | 100 | MN161213 |
|  | L-2278 | *Flavobacterium* sp. | Negative | 99 | Subglacial ice | Svalbard - Ny Alesund | 2017 | 15 | 20 | MK670521 |
|  | L-2280 | *Flavobacterium sp.* | Negative | 99 | Subglacial ice | Svalbard - Ny Alesund | 2017 | 15 | 5 | MK670533 |
|  | L-2282 | *Flavobacterium sp.* | Negative | 99 | Subglacial ice | Svalbard - Ny Alesund | 2017 | 15 | 5 | MK670527 |
|  | L-2284 | *Flavobacterium sp.* | Negative | 99 | Subglacial ice | Svalbard - Ny Alesund | 2017 | 15 | 5 | MK670535 |
|  | L-2291 | *Flavobacterium sp.* | Negative | 99 | Subglacial ice | Svalbard - Ny Alesund | 2017 | 15 | 5 | MK670552 |
|  | L-2292 | *Flavobacterium sp.* | Negative | 99 | Subglacial ice | Svalbard - Ny Alesund | 2017 | 15 | 5 | MK670553 |
|  | L-2293 | *Flavobacterium sp.* | Negative | 99 | Subglacial ice | Svalbard - Ny Alesund | 2017 | 15 | 50 | MK670549 |
|  | L-2560 | *Flavobacterium sp.* | Negative | 99 | Subglacial ice | Svalbard - Ny Alesund | 2017 | 15 | 5 | MK670522 |
|  | L-2561 | *Flavobacterium sp.* | Negative | 99 | Subglacial ice | Svalbard - Ny Alesund | 2017 | 15 | 10 | MK670518 |
|  | L-2562 | *Flavobacterium sp.* | Negative | 99 | Subglacial ice | Svalbard - Ny Alesund | 2017 | 15 | 10 | MK670519 |
|  | L-2563 | *Flavobacterium sp.* | Negative | 100 | Subglacial ice | Svalbard - Ny Alesund | 2017 | 15 | 20 | MK670520 |
|  | L-2567 | *Flavobacterium sp.* | Negative | 99 | Subglacial ice | Svalbard - Ny Alesund | 2017 | 15 | 5 | MK670529 |
|  | L-2572 | *Flavobacterium sp.* | Negative | 99 | Subglacial ice | Svalbard - Ny Alesund | 2017 | 15 | 5 | MK670540 |
|  | L-2573 | *Flavobacterium sp.* | Negative | 99 | Subglacial ice | Svalbard - Ny Alesund | 2017 | 15 | 5 | MK670541 |
|  | L-2574 | *Flavobacterium sp.* | Negative | 99 | Subglacial ice | Svalbard - Ny Alesund | 2017 | 15 | 5 | MK670542 |
|  | L-2662 | *Flavobacterium sp.* | Negative | 99 | Subglacial ice | Svalbard - Ny Alesund | 2017 | 15 | 5 | MK670523 |
|  | L-1994 | *Flavobacterium sp.* | Negative | 96 | Tap water | Svalbard - Longyearbyen | 2016 | 37 | 1000 | MN161215 |
|  | N72 | *Flavobacterium* sp. | Negative | 98 | Soil | Svalbard - Ny Alesund | 2017 | 5 | n/a | MH714638 |
|  | L-2564 | *Hymenobacter* sp. | Negative | 99 | Subglacial ice | Svalbard - Ny Alesund | 2017 | 15 | 5 | MK670531 |
|  | N36a | *Pedobacter sp.* | Negative | 99 | Sediment | Svalbard - Ny Alesund | 2017 | 17 | n/a | MH714626 |
|  | L-1969 | *Pedobacter sp.* | Negative | 99 | Lake ice | Svalbard - Longyearbyen | 2016 | 10 | 125 | MN161209 |
|  | L-1973 | *Pedobacter sp.* | Negative | 99 | Lake ice | Svalbard - Longyearbyen | 2016 | 10 | 50 | MN161211 |
|  | L-2566 | *Spirosoma* sp. | Negative | 99 | Subglacial ice | Svalbard - Ny Alesund | 2017 | 15 | 5 | MK670528 |
| Firmicutes | N10a | *Bacillus* sp. | Positive | 99 | Soil | Svalbard - Ny Alesund | 2017 | 37 | n/a | MH714615 |
|  | N10b | *Bacillus* sp. | Positive | 99 | Soil | Svalbard - Ny Alesund | 2017 | 37 | n/a | MN450707 |
|  | N17 | *Bacillus* sp. | Positive | 99 | Soil | Svalbard - Ny Alesund | 2017 | 37 | n/a | MH714617 |
|  | N21 | *Bacillus* sp. | Positive | 98 | Sediment | Svalbard - Ny Alesund | 2017 | 37 | n/a | MN450710 |
|  | N22 | *Bacillus* sp. | Positive | 99 | Sediment | Svalbard - Ny Alesund | 2017 | 37 | n/a | MN450711 |
|  | N23 | *Bacillus* sp. | Positive | 99 | Sediment | Svalbard - Ny Alesund | 2017 | 37 | n/a | MH714619 |
|  | N24 | *Bacillus* sp. | Positive | 83 | Sediment | Svalbard - Ny Alesund | 2017 | 37 | n/a | MH714620 |
|  | N3 | *Bacillus* sp. | Positive | 99 | Soil | Svalbard - Ny Alesund | 2017 | 37 | n/a | MN450700 |
|  | N34 | *Bacillus* sp. | Positive | 99 | Soil | Svalbard - Ny Alesund | 2017 | 37 | n/a | MH714624 |
|  | N4 | *Bacillus* sp. | Positive | 99 | Soil | Svalbard - Ny Alesund | 2017 | 37 | n/a | MN450701 |
|  | N4b | *Bacillus* sp. | Positive | 99 | Soil | Svalbard - Ny Alesund | 2017 | 37 | n/a | MN450702 |
|  | N5 | *Bacillus* sp. | Positive | 99 | Soil | Svalbard - Ny Alesund | 2017 | 37 | n/a | MN450703 |
|  | N5a | *Bacillus* sp. | Positive | 96 | Soil | Svalbard - Ny Alesund | 2017 | 37 | n/a | MN450704 |
|  | 12396_bac | *Bacillus* sp. | Positive | 100 | Clear ice | Greenland Ice Sheet | 2017 | 15 | 100 | MK453054 |
|  | L-1918 | *Bacillus* sp. | Positive | 99 | Clear ice | Greenland Ice Sheet | 2016 | 15 | 0.1 | MK453120 |
|  | L-1919 | *Bacillus* sp. | Positive | 99 | Clear ice | Greenland Ice Sheet | 2016 | 15 | 0.1 | MK453121 |
|  | L-1920 | *Bacillus* sp. | Positive | 99 | Clear ice | Greenland Ice Sheet | 2016 | 15 | 0.1 | MK453122 |
|  | L-1921 | *Bacillus* sp. | Positive | 100 | Clear ice | Greenland Ice Sheet | 2016 | 15 | 0.1 | MK453123 |
|  | L-1923 | *Bacillus* sp. | Positive | 99 | Clear ice | Greenland Ice Sheet | 2016 | 15 | 0.1 | MK453125 |
|  | L-1924 | *Bacillus* sp. | Positive | 99 | Clear ice | Greenland Ice Sheet | 2016 | 15 | 0.1 | MK453126 |
|  | L-1925 | *Bacillus* sp. | Positive | 99 | Clear ice | Greenland Ice Sheet | 2016 | 15 | 0.1 | MK453124 |
|  | L-1905 | *Bacillus* sp. | Positive | 99 | Cryoconite | Greenland Ice Sheet | 2016 | 15 | 0.1 | MK453108 |
|  | L-1912 | *Bacillus* sp. | Positive | 99 | Cryoconite | Greenland Ice Sheet | 2016 | 15 | 0.1 | MK453114 |
|  | L-1904 | *Bacillus* sp. | Positive | 99 | Cryoconite | Greenland Ice Sheet | 2016 | 15 | 0.1 | MK453107 |
|  | L-1913 | *Bacillus* sp. | Positive | 99 | Cryoconite | Greenland Ice Sheet | 2016 | 15 | 0.1 | MK453115 |
|  | L-1914 | *Bacillus* sp. | Positive | 99 | Cryoconite | Greenland Ice Sheet | 2016 | 15 | 0.1 | MK453116 |
|  | L-1915 | *Bacillus* sp. | Positive | 99 | Cryoconite | Greenland Ice Sheet | 2016 | 15 | 0.1 | MK453117 |
|  | L-1916 | *Bacillus* sp. | Positive | 99 | Cryoconite | Greenland Ice Sheet | 2016 | 15 | 0.1 | MK453118 |
|  | L-1895 | *Bacillus* sp. | Positive | 99 | Cryoconite | Greenland Ice Sheet | 2016 | 15 | 0.1 | MK453100 |
|  | L-1896 | *Bacillus* sp. | Positive | 100 | Cryoconite | Greenland Ice Sheet | 2016 | 15 | 0.1 | MK453101 |
|  | L-1897 | *Bacillus* sp. | Positive | 99 | Cryoconite | Greenland Ice Sheet | 2016 | 15 | 0.1 | MK453102 |
|  | 12403-Bac | *Bacillus* sp. | Positive | 100 | Dark ice | Greenland Ice Sheet | 2017 | 15 | 0.1 | MK453055 |
|  | L-1910 | *Bacillus* sp. | Positive | 100 | Dispersed cryoconite | Greenland Ice Sheet | 2016 | 15 | 0.1 | MK453112 |
|  | L-1906 | *Bacillus* sp. | Positive | 100 | Dispersed cryoconite | Greenland Ice Sheet | 2016 | 15 | 0.1 | MK453109 |
|  | L-1907 | *Bacillus* sp. | Positive | 100 | Dispersed cryoconite | Greenland Ice Sheet | 2016 | 15 | 0.1 | MK453110 |
|  | L-1917 | *Bacillus* sp. | Positive | 100 | Dispersed cryoconite | Greenland Ice Sheet | 2016 | 15 | 0.1 | MK453119 |
|  | N8 | *Bacillus* sp. | Positive | 93 | Soil | Svalbard - Ny Alesund | 2017 | 37 | n/a | MN450706 |
|  | N83 | *Bacillus* sp. | Positive | 99 | Snow | Svalbard - Ny Alesund | 2017 | 17 | 3000 | MH714642 |
|  | N85 | *Bacillus* sp. | Positive | 98 | Melt water | Svalbard - Ny Alesund | 2017 | 37 | 2000 | MH714644 |
|  | N93 | *Bacillus* sp. | Positive | 99 | Sea water | Svalbard - Ny Alesund | 2017 | 37 | 50 | MH714648 |
|  | N95 | *Bacillus* sp. | Positive | 88 | Sediment | Svalbard - Ny Alesund | 2017 | 37 | n/a | MN450729 |
|  | S37 | *Bacillus* sp. | Positive | 99 | Sediment | Svalbard - Longyearbyen | 2016 | 37 | n/a | MN450689 |
|  | S39 | *Bacillus* sp. | Positive | 99 | Snow | Svalbard - Longyearbyen | 2016 | 5 | 50 | MN450690 |
|  | S44 | *Bacillus* sp. | Positive | 99 | Snow | Svalbard - Longyearbyen | 2016 | 37 | 50 | MH714670 |
|  | S64 | *Bacillus* sp. | Positive | 99 | Sediment | Svalbard - Longyearbyen | 2016 | 37 | n/a | MN450694 |
|  | S7 | *Bacillus* sp. | Positive | 99 | Sediment | Svalbard - Longyearbyen | 2016 | 37 | n/a | MH714653 |
|  | S70 | *Bacillus* sp. | Positive | 99 | Sediment | Svalbard - Longyearbyen | 2016 | 37 | n/a | MH714676 |
|  | S71 | *Bacillus* sp. | Positive | 99 | Sediment | Svalbard - Longyearbyen | 2016 | 37 | n/a | MH714677 |
|  | S80 | *Bacillus* sp. | Positive | 99 | Sediment | Svalbard - Longyearbyen | 2016 | 17 | n/a | MN450699 |
|  | N39 | *Carnobacterium* sp. | Positive | 99 | Sediment | Svalbard - Ny Alesund | 2017 | 17 | n/a | MH714627 |
|  | N39a | *Carnobacterium* sp. | Positive | 99 | Sediment | Svalbard - Ny Alesund | 2017 | 17 | n/a | MN450716 |
|  | N58 | *Carnobacterium* sp. | Positive | 99 | Sediment | Svalbard - Ny Alesund | 2017 | 5 | n/a | MH714634 |
|  | S27b | *Carnobacterium* sp. | Positive | 99 | Sediment | Svalbard - Longyearbyen | 2016 | 17 | n/a | MH714664 |
|  | An58 | *Enterococcus* sp. | Positive | 99 | Sediment | Svalbard - Longyearbyen | 2016 | 17 | n/a | MH714608 |
|  | N11 | *Exiguobacterium* sp. | Positive | 99 | Sediment | Svalbard - Ny Alesund | 2017 | 37 | n/a | MN450708 |
|  | N19 | *Exiguobacterium* sp. | Positive | 99 | Soil | Svalbard - Ny Alesund | 2017 | 37 | n/a | MN450709 |
|  | N2 | *Exiguobacterium* sp. | Positive | 99 | Soil | Svalbard - Ny Alesund | 2017 | 37 | n/a | MH714612 |
|  | N29 | *Exiguobacterium* sp. | Positive | 95 | Soil | Svalbard - Ny Alesund | 2017 | 17 | n/a | MN450712 |
|  | N37 | *Exiguobacterium* sp. | Positive | 99 | Sediment | Svalbard - Ny Alesund | 2017 | 17 | n/a | MN450714 |
|  | N44 | *Exiguobacterium* sp. | Positive | 99 | Sediment | Svalbard - Ny Alesund | 2017 | 37 | n/a | MN450718 |
|  | N45 | *Exiguobacterium* sp. | Positive | 99 | Sediment | Svalbard - Ny Alesund | 2017 | 37 | n/a | MN450719 |
|  | N57 | *Exiguobacterium* sp. | Positive | 99 | Soil | Svalbard - Ny Alesund | 2017 | 37 | n/a | MN450723 |
|  | N9 | *Exiguobacterium* sp. | Positive | 99 | Soil | Svalbard - Ny Alesund | 2017 | 37 | n/a | MH714614 |
|  | S1 | *Fictibacillus* sp. | Positive | 99 | Sediment | Svalbard - Longyearbyen | 2016 | 37 | n/a | MN450684 |
|  | S5 | *Fictibacillus* sp. | Positive | 100 | Sea water | Svalbard - Longyearbyen | 2016 | 37 | 50 | MH714652 |
|  | N81b | *Lysinibacillus* sp. | Positive | 99 | Snow | Svalbard - Ny Alesund | 2017 | 17 | 3000 | MH714640 |
|  | S77 | *Lysinibacillus* sp. | Positive | 99 | Sediment | Svalbard - Longyearbyen | 2016 | 37 | n/a | MH714680 |
|  | S77a | *Lysinibacillus* sp. | Positive | 99 | Sediment | Svalbard - Longyearbyen | 2016 | 37 | n/a | MN450698 |
|  | L-2661 | *Paenibacillus* *antarcticus* | Positive | 100 | Subglacial ice | Svalbard - Ny Alesund | 2017 | 15 | 5 | MK670537 |
|  | N57b | *Paenibacillus* sp. | Positive | 99 | Soil | Svalbard - Ny Alesund | 2017 | 37 | n/a | MH714633 |
|  | N7 | *Paenibacillus* sp. | Positive | 99 | Soil | Svalbard - Ny Alesund | 2017 | 37 | n/a | MH714613 |
|  | N82 | *Paenibacillus* sp. | Positive | 99 | Snow | Svalbard - Ny Alesund | 2017 | 17 | 3000 | MH714641 |
|  | An28 | *Psychrobacillus* sp. | Positive | 93 | Snow | Svalbard - Longyearbyen | 2016 | 17 | 50 | MN450679 |
|  | N54 | *Psychrobacillus* sp. | Positive | 94 | Soil | Svalbard - Ny Alesund | 2017 | 37 | n/a | MH714632 |
|  | N6 | *Psychrobacillus* sp. | Positive | 94 | Sediment | Svalbard - Ny Alesund | 2017 | 37 | n/a | MN450705 |
|  | S33 | *Psychrobacillus* sp. | Positive | 94 | Sea water | Svalbard - Longyearbyen | 2016 | 17 | 50 | MN450688 |
|  | S69 | *Psychrobacillus* sp. | Positive | 94 | Sediment | Svalbard - Longyearbyen | 2016 | 17 | n/a | MN450696 |
|  | N36 | *Sporosarcina* sp. | Positive | 99 | Sediment | Svalbard - Ny Alesund | 2017 | 17 | n/a | MH714625 |
|  | N38 | *Sporosarcina* sp. | Positive | 99 | Sediment | Svalbard - Ny Alesund | 2017 | 17 | n/a | MN450715 |
|  | S15 | *Sporosarcina* sp. | Positive | 99 | Sediment | Svalbard - Longyearbyen | 2016 | 17 | n/a | MH714656 |
|  | S90 | *Sporosarcina* sp. | Positive | 98 | Sediment | Svalbard - Longyearbyen | 2016 | 17 | n/a | MH714685 |
| Proteobacteria | L-2655 | *Halomonas* sp. | Negative | 96 | Dark ice | Greenland Ice Sheet | 2016 | 15 | 0.1 | MN161221 |
|  | L-2288 | *Herminiimonas* sp. | Negative | 99 | Subglacial ice | Svalbard - Ny Alesund | 2017 | 15 | 5 | MK670550 |
|  | L-2290 | *Herminiimonas* sp. | Negative | 99 | Subglacial ice | Svalbard - Ny Alesund | 2017 | 15 | 5 | MK670551 |
|  | L-1993 | *Janthinobacterium lividum* | Negative | 100 | Tap water | Svalbard - Longyearbyen | 2016 | 37 | 1000 | - |
|  | L-2266 | *Janthinobacterium* sp. | Negative | 99 | Cryoconite | Greenland Ice Sheet | 2017 | 15 | 10 | MK453066 |
|  | L-2294 | *Janthinobacterium* sp. | Negative | 99 | Cryoconite | Greenland Ice Sheet | 2017 | 15 | 10 | MK453067 |
|  | L-1995 | *Janthinobacterium* sp. | Negative | 99 | Tap water | Svalbard - Longyearbyen | 2016 | 37 | 1000 | MN161216 |
|  | L-2271 | *Massilia* sp. | Negative | 100 | Dark ice | Greenland Ice Sheet | 2016 | 15 | 0.1 | MN161219 |
|  | L-2555 | *Massilia* sp. | Negative | 99 | Glacial meltwater | Svalbard - Ny Alesund | 2017 | 15 | 50 | - |
|  | L-2653 | *Massilia* sp. | Negative | 99 | Glacial meltwater | Svalbard - Ny Alesund | 2017 | 15 | 10 | MK670506 |
|  | L-2283 | *Massilia* sp. | Negative | 99 | Subglacial ice | Svalbard - Ny Alesund | 2017 | 15 | 20 | MK670525 |
|  | L-2577 | *Massilia* sp. | Negative | 99 | Subglacial ice | Svalbard - Ny Alesund | 2017 | 15 | 20 | MK670545 |
|  | L-2692 | *Massilia* sp. | Negative | 99 | Subglacial ice | Svalbard - Ny Alesund | 2017 | 15 | 5 | MK670538 |
|  | L-2272 | *Massilia* sp. | Negative | 99 | Supraglacial water | Greenland Ice Sheet | 2017 | 15 | 100 | MK453070 |
|  | L-2275 | *Massilia* sp. | Negative | 99 | Supraglacial water | Greenland Ice Sheet | 2017 | 15 | 100 | MK453068 |
|  | L-2276 | *Massilia* sp. | Negative | 99 | Supraglacial water | Greenland Ice Sheet | 2017 | 15 | 100 | MK453069 |
|  | L-2558 | *Polaromonas* sp. | Negative | 99 | Glacial meltwater | Svalbard - Ny Alesund | 2017 | 15 | 10 | MK670510 |
|  | L-2565 | *Polaromonas* sp. | Negative | 100 | Subglacial ice | Svalbard - Ny Alesund | 2017 | 15 | 5 | MK670532 |
|  | N92 | *Polymorphobacter* sp. | Negative | 99 | Pond water | Svalbard - Ny Alesund | 2017 | 17 | 1000 | MH714647 |
|  | L-2660 | *Pseudomonas fluorescens* | Negative | 100 | Dark ice | Greenland Ice Sheet | 2016 | 15 | 0.1 | - |
|  | L-2644 | *Pseudomonas fluorescens* | Negative | 99 | Dark ice | Greenland Ice Sheet | 2016 | 15 | 0.1 | MN161220 |
|  | L-2656 | *Pseudomonas fluorescens* | Negative | 100 | Dark ice | Greenland Ice Sheet | 2016 | 15 | 0.1 | MN161222 |
|  | L-2658 | *Pseudomonas fluorescens* | Negative | 99 | Dark ice | Greenland Ice Sheet | 2016 | 15 | 0.1 | MN161224 |
|  | L-2652 | *Pseudomonas frederiksbergensis* | Negative | 100 | Glacial meltwater | Svalbard - Ny Alesund | 2017 | 15 | 10 | MK670511 |
|  | L-2553 | *Pseudomonas graminis* | Negative | 100 | Glacial meltwater | Svalbard - Ny Alesund | 2017 | 15 | 10 | MK670514 |
|  | L-2646 | *Pseudomonas graminis* | Negative | 100 | Glacial meltwater | Svalbard - Ny Alesund | 2017 | 15 | 10 | MK670504 |
|  | L-1974 | *Pseudomonas graminis* | Negative | 100 | Lake ice | Svalbard - Longyearbyen | 2016 | 10 | 50 | - |
|  | L-2570 | *Pseudomonas graminis* | Negative | 100 | Subglacial ice | Svalbard - Ny Alesund | 2017 | 15 | 50 | MK670524 |
|  | N15 | *Pseudomonas* sp. | Negative | 99 | Soil | Svalbard - Ny Alesund | 2017 | 17 | n/a | MH714616 |
|  | N30b | *Pseudomonas* sp. | Negative | 98 | Soil | Svalbard - Ny Alesund | 2017 | 17 | n/a | MH714623 |
|  | N40 | *Pseudomonas* sp. | Negative | 98 | Soil | Svalbard - Ny Alesund | 2017 | 5 | n/a | MN450717 |
|  | N50 | *Pseudomonas* sp. | Negative | 97 | Soil | Svalbard - Ny Alesund | 2017 | 5 | n/a | MN450720 |
|  | N60 | *Pseudomonas* sp. | Negative | 98 | Soil | Svalbard - Ny Alesund | 2017 | 5 | n/a | MH714635 |
|  | N71 | *Pseudomonas* sp. | Negative | 99 | Soil | Svalbard - Ny Alesund | 2017 | 5 | n/a | MH714637 |
|  | N87 | *Pseudomonas* sp. | Negative | 98 | Sea water | Svalbard - Ny Alesund | 2017 | 5 | 50 | MH714645 |
|  | S67 | *Pseudomonas* sp. | Negative | 99 | Sediment | Svalbard - Longyearbyen | 2016 | 17 | n/a | MH714675 |
|  | L-2145 | *Pseudomonas* sp. | Negative | 100 | Cryoconite | Greenland Ice Sheet | 2017 | 15 | 10 | MK453079 |
|  | L-2144 | *Pseudomonas* sp. | Negative | 99 | Cryoconite | Greenland Ice Sheet | 2017 | 15 | 10 | MK453080 |
|  | L-2643 | *Pseudomonas* sp. | Negative | 99 | Cryoconite | Greenland Ice Sheet | 2017 | 15 | 10 | MK453084 |
|  | L-2268 | *Pseudomonas* sp. | Negative | 99 | Cryoconite | Greenland Ice Sheet | 2017 | 15 | 10 | MK453077 |
|  | L-2265 | *Pseudomonas* sp. | Negative | 100 | Cryoconite | Greenland Ice Sheet | 2017 | 15 | 10 | MK453078 |
|  | L-2696 | *Pseudomonas* sp. | Negative | 99 | Cryoconite | Greenland Ice Sheet | 2017 | 15 | 10 | MK453081 |
|  | L-2137 | *Pseudomonas* sp. | Negative | 100 | Cryoconite | Greenland Ice Sheet | 2017 | 15 | 10 | MK453082 |
|  | L-2142 | *Pseudomonas* sp. | Negative | 99 | Cryoconite | Greenland Ice Sheet | 2017 | 15 | 10 | MK453083 |
|  | L-2657 | *Pseudomonas* sp. | Negative | 99 | Dark ice | Greenland Ice Sheet | 2016 | 15 | 0.1 | MN161223 |
|  | L-2659 | *Pseudomonas* sp. | Negative | 99 | Dark ice | Greenland Ice Sheet | 2016 | 15 | 0.1 | MN161225 |
|  | L-2554 | *Pseudomonas* sp. | Negative | 100 | Glacial meltwater | Svalbard - Ny Alesund | 2017 | 15 | 10 | MK670515 |
|  | L-2557 | *Pseudomonas* sp. | Negative | 99 | Glacial meltwater | Svalbard - Ny Alesund | 2017 | 15 | 10 | MK670508 |
|  | L-2650 | *Pseudomonas* sp. | Negative | 99 | Glacial meltwater | Svalbard - Ny Alesund | 2017 | 15 | 50 | MK670512 |
|  | L-1964 | *Pseudomonas* sp. | Negative | 99 | Lake ice | Svalbard - Longyearbyen | 2016 | 10 | 100 | MN161207 |
|  | L-1976 | *Pseudomonas* sp. | Negative | 99 | Lake ice | Svalbard - Longyearbyen | 2016 | 10 | 100 | - |
|  | L-1978 | *Pseudomonas* sp. | Negative | 99 | Sea water | Svalbard - Longyearbyen | 2016 | 10 | 100 | - |
|  | L-1979 | *Pseudomonas* sp. | Negative | 99 | Sea water | Svalbard - Longyearbyen | 2016 | 10 | 100 | - |
|  | L-1982 | *Pseudomonas* sp. | Negative | 99 | Sea water | Svalbard - Longyearbyen | 2016 | 10 | 100 | - |
|  | L-1983 | *Pseudomonas* sp. | Negative | 99 | Sea water | Svalbard - Longyearbyen | 2016 | 10 | 100 | MN161214 |
|  | L-2575 | *Pseudomonas* sp. | Negative | 100 | Subglacial ice | Svalbard - Ny Alesund | 2017 | 15 | 5 | MK670547 |
|  | L-2578 | *Pseudomonas* sp. | Negative | 100 | Subglacial ice | Svalbard - Ny Alesund | 2017 | 15 | 20 | MK670546 |
|  | S88 | *Pseudomonas* sp. | Negative | 99 | Sediment | Svalbard - Longyearbyen | 2016 | 17 | n/a | MH714684 |
|  | N61 | *Psychrobacter* sp. | Negative | 99 | Sediment | Svalbard - Ny Alesund | 2017 | 17 | n/a | MH714636 |
|  | S35 | *Psychrobacter* sp. | Negative | 99 | Sediment | Svalbard - Longyearbyen | 2016 | 5 | n/a | MH714669 |
|  | S84 | *Psychrobacter* sp. | Negative | 99 | Sediment | Svalbard - Longyearbyen | 2016 | 5 | n/a | MH714682 |
|  | L-2695 | *Rahnella* sp. | Negative | 99 | Dark ice | Greenland Ice Sheet | 2016 | 15 | 0.1 | MN161227 |
|  | L-1980 | *Raoultella* sp. | Negative | 99 | Sea water | Svalbard - Longyearbyen | 2016 | 10 | 100 | MN161212 |
|  | L-1894 | *Rhodopseudomonas* sp. | Negative | 99 | Cryoconite | Greenland Ice Sheet | 2016 | 15 | 0.1 | MK453099 |
|  | N78 | *Roseomonas* sp. | Negative | 99 | Soil | Svalbard - Ny Alesund | 2017 | 17 | n/a | MH714639 |
|  | N96 | *Roseomonas* sp. | Negative | 97 | Sediment | Svalbard - Ny Alesund | 2017 | 17 | n/a | MH714649 |
|  | L-2135 | *Rugamonas rubra* | Negative | 100 | Clear ice | Greenland Ice Sheet | 2017 | 15 | 100 | MK453088 |
|  | L-2139 | *Rugamonas rubra* | Negative | 100 | Cryoconite | Greenland Ice Sheet | 2017 | 15 | 0.1 | MK453085 |
|  | L-2140 | *Rugamonas rubra* | Negative | 100 | Cryoconite | Greenland Ice Sheet | 2017 | 15 | 0.1 | MK453086 |
|  | L-2141 | *Rugamonas rubra* | Negative | 100 | Cryoconite | Greenland Ice Sheet | 2017 | 15 | 0.1 | MK453087 |
|  | L-2130 | *Rugamonas rubra* | Negative | 100 | Dark ice | Greenland Ice Sheet | 2017 | 15 | 10 | MK453090 |
|  | N30 | *Rugamonas* sp. | Negative | 98 | Soil | Svalbard - Ny Alesund | 2017 | 17 | n/a | MH714622 |
|  | L-2648 | *Sphingomonas faeni* | Negative | 100 | Glacial meltwater | Svalbard - Ny Alesund | 2017 | 15 | 10 | MK670516 |
|  | L-2131 | *Sphingomonas glacialis* | Negative | 100 | Dark ice | Greenland Ice Sheet | 2017 | 15 | 10 | MK453091 |
|  | L-2649 | *Sphingomonas glacialis* | Negative | 100 | Glacial meltwater | Svalbard - Ny Alesund | 2017 | 15 | 10 | MK670517 |
|  | N43 | *Sphingomonas* sp. | Negative | 99 | Soil | Svalbard - Ny Alesund | 2017 | 17 | n/a | MH714630 |
|  | L-2552 | *Sphingomonas* sp. | Negative | 100 | Cryoconite | Greenland Ice Sheet | 2017 | 15 | 0.1 | MK453092 |
|  | L-1900 | *Sphingomonas* sp. | Negative | 99 | Cryoconite | Greenland Ice Sheet | 2016 | 15 | 0.1 | MK453105 |
|  | L-1901 | *Sphingomonas* sp. | Negative | 99 | Cryoconite | Greenland Ice Sheet | 2016 | 15 | 0.1 | MK453106 |
|  | L-1902 | *Sphingomonas* sp. | Negative | 99 | Cryoconite | Greenland Ice Sheet | 2016 | 15 | 0.1 | - |
|  | L-1972 | *Sphingomonas* sp. | Negative | 100 | Lake ice | Svalbard - Longyearbyen | 2016 | 10 | 50 | MN161210 |
|  | L-2694 | *Sphingomonas* sp. | Negative | 99 | Snow | Greenland Ice Sheet | 2017 | 15 | 100 | MN161226 |
|  | L-2273 | *Sphingomonas* sp. | Negative | 100 | Supraglacial water | Greenland Ice Sheet | 2017 | 15 | 100 | MK453093 |
|  | L-2651 | *Sphingorhabdus* sp. | Negative | 100 | Glacial meltwater | Svalbard - Ny Alesund | 2017 | 15 | 10 | MK670509 |
|  | L-2267 | *Undibacterium* sp. | Negative | 99 | Cryoconite | Greenland Ice Sheet | 2017 | 15 | 0.1 | MK453095 |
|  | L-2147 | *Undibacterium* sp. | Negative | 99 | Cryoconite | Greenland Ice Sheet | 2017 | 15 | 0.1 | - |
|  | L-2269 | *Undibacterium* sp. | Negative | 99 | Cryoconite | Greenland Ice Sheet | 2017 | 15 | 10 | MK453098 |
|  | L-2138 | *Undibacterium* sp. | Negative | 99 | Cryoconite | Greenland Ice Sheet | 2017 | 15 | 0.1 | MK453096 |
|  | L-2143 | *Undibacterium* sp. | Negative | 99 | Cryoconite | Greenland Ice Sheet | 2017 | 15 | 0.1 | MK453097 |
|  | L-2059 | *Undibacterium* sp. | Negative | 99 | Dark ice | Greenland Ice Sheet | 2016 | 15 | 0.1 | - |
|  | L-2060 | *Undibacterium* sp. | Negative | 99 | Dark ice | Greenland Ice Sheet | 2016 | 15 | 0.1 | - |
|  | L-2063 | *Undibacterium* sp. | Negative | 99 | Dark ice | Greenland Ice Sheet | 2016 | 15 | 0.1 | MN161218 |
|  | L-2647 | unidentified Burkholderiaceae | Negative | 99 | Glacial meltwater | Svalbard - Ny Alesund | 2017 | 15 | 10 | MK670505 |
|  | L-2432 | unidentified Oxalobacteraceae | Negative | 98 | Clear ice | Greenland Ice Sheet | 2017 | 15 | 100 | MK453094 |
|  | L-2146 | unidentified Oxalobacteraceae | Negative | 96 | Cryoconite | Greenland Ice Sheet | 2017 | 15 | 0.1 | MK453089 |
|  | L-2270 | unidentified Oxalobacteraceae | Negative | 97 | Cryoconite | Greenland Ice Sheet | 2017 | 15 | 0.1 | MK453071 |
|  | L-2132 | unidentified Oxalobacteraceae | Negative | 98 | Dark ice | Greenland Ice Sheet | 2017 | 15 | 10 | MK453073 |
|  | L-2286 | unidentified Oxalobacteraceae | Negative | 98 | Subglacial ice | Svalbard - Ny Alesund | 2017 | 15 | 5 | MK670536 |
|  | L-2569 | unidentified Oxalobacteraceae | Negative | 98 | Subglacial ice | Svalbard - Ny Alesund | 2017 | 15 | 50 | MK670526 |
|  | L-2581 | unidentified Oxalobacteraceae | Negative | 98 | Subglacial ice | Svalbard - Ny Alesund | 2017 | 15 | 20 | MK670548 |
|  | L-2274 | unidentified Oxalobacteraceae | Negative | 98 | Supraglacial water | Greenland Ice Sheet | 2017 | 15 | 0.1 | MK453075 |
|  | L-2428 | unidentified Oxalobacteraceae | Negative | 98 | Supraglacial water | Greenland Ice Sheet | 2017 | 15 | 0.1 | MK453074 |
|  | L-2546 | unidentified Oxalobacteraceae | Negative | 98 | Supraglacial water | Greenland Ice Sheet | 2017 | 15 | 0.1 | MK453076 |
|  | L-2547 | unidentified Oxalobacteraceae | Negative | 98 | Supraglacial water | Greenland Ice Sheet | 2017 | 15 | 0.1 | MK453072 |

**Supplementary table S2. Isolates used for growth at body temperature (37°C), hemolytic activity and antimicrobial susceptibility testing. EXB: collection number. Growth at 37°C: + positive growth, (+) moderate growth, (-) limited growth, - no growth.**

| Phylum | Isolate (EXB) | Species | Gram stain | Id. % closest neighbor | Sample | Sampling location | Sampling year | Isolation temp. (°C) | Growth at 37C | Filtrated volume (ml) | Genbank accession number |
| --- | --- | --- | --- | --- | --- | --- | --- | --- | --- | --- | --- |
| ACTINOBACTERIA | L-1909 | *Arthrobacter sp.* | Positive | 100 | Dispersed cryoconite | Greenland | 2016 | 15 | (+) | 0.1 | MK453111 |
|  | S24 (L-5712) | Brevibacterium | Positive | 99 | Sediment | Svalbard - Longyearbyen | 2016 | 17 | + | n/a | MH714661 |
|  | L-1899 | *Cryobacterium psychrotolerans* | Positive | 100 | Cryoconite | Greenland | 2016 | 15 | (-) | 0.1 | MK453104 |
|  | L-2062 | *Cryobacterium sp.* | Positive | 100 | Dark ice | Greenland | 2016 | 15 | (+) | 0.1 | MN161217 |
|  | L-2263 | *Cryobacterium* sp. | Positive | 100 | Clear ice | Greenland | 2017 | 15 | - | 100 | MK453057 |
|  | L-2279 | *Cryobacterium* sp. | Positive | 97 | Subglacial ice | Svalbard | 2017 | 15 | - | 5 | - |
|  | L-2285 | *Cryobacterium* sp. | Positive | 99 | Subglacial ice | Svalbard | 2017 | 15 | (-) | 5 | MK670539 |
|  | L-2571 | *Cryobacterium* sp. | Positive | 100 | Subglacial ice | Svalbard | 2017 | 15 | (+) | 5 | MK670534 |
|  | L-2580 | *Cryobacterium* sp. | Positive | 99 | Subglacial ice | Svalbard | 2017 | 15 | (-) | 5 | MK670544 |
|  | L-2264 | *Curtobacterium* sp. | Positive | 100 | Snow | Greenland | 2017 | 15 | + | 100 | MK453059 |
|  | L-2433 | *Curtobacterium* sp. | Positive | 100 | Dark ice | Greenland | 2017 | 15 | + | 0.1 | MK453060 |
|  | L-2430 | *Frigoribacterium* sp. | Positive | 99 | Supraglacial water | Greenland | 2017 | 15 | (-) | 0.1 | MK453065 |
|  | An34 (L-5721) | Leifsonia | Positive | 98 | Sea water | Svalbard - Longyearbyen | 2016 | 5 | (-) | 50 | MH714607 |
|  | S8 (L-5709) | Microbacterium | Positive | 99 | Sediment | Svalbard - Longyearbyen | 2016 | 37 | + | n/a | MH714654 |
|  | S23b (L-5711) | Microbacterium | Positive | 98 | Sea water | Svalbard - Longyearbyen | 2016 | 37 | + | 50 | MH714660 |
|  | S26 (L-5713) | Microbacterium | Positive | 98 | Sediment | Svalbard - Longyearbyen | 2016 | 17 | (-) | n/a | MH714663 |
|  | S60 (L-5718) | Microbacterium | Positive | 98 | Sea water | Svalbard - Longyearbyen | 2016 | 5 | + | 50 | MH714673 |
|  | L-1922 | *Micrococcus sp.* | Positive | 99 | Clear ice | Greenland | 2016 | 15 | + | 0.1 | MK453127 |
|  | S3 (L-5707) | Micromonospora | Positive | 99 | Sediment | Svalbard - Longyearbyen | 2016 | 37 | + | n/a | MH714651 |
|  | N18 (L-5691) | Micromonospora | Positive | 99 | Soil | Svalbard - Ny Alesund | 2017 | 37 | + | n/a | MH714618 |
|  | S10 (L-5710) | Oerskovia | Positive | 99 | Sea water | Svalbard - Longyearbyen | 2016 | 37 | (+) | 50 | MH714655 |
|  | S58 (L-5717) | Salinibacterium | Positive | 99 | Sea water | Svalbard - Longyearbyen | 2016 | 5 | + | 50 | MH714672 |
|  | N106 (L-5706) | Salinibacterium | Positive | 99 | Melt water | Svalbard - Ny Alesund | 2017 | 17 | (+) | 2000 | MH714650 |
|  | N28 (L-5694) | Streptomyces | Positive | 99 | Soil | Svalbard - Ny Alesund | 2017 | 17 | (+) | n/a | MH714621 |
|  | N41 (L-5699) | Streptomyces | Positive | 99 | Soil | Svalbard - Ny Alesund | 2017 | 17 | - | n/a | MH714628 |
|  | N42 (L-5700) | Streptomyces | Positive | 99 | Soil | Svalbard - Ny Alesund | 2017 | 17 | (+) | n/a | MH714629 |
|  | S32 (L-5715) | Tessaracoccus | Positive | 99 | Sea water | Svalbard - Longyearbyen | 2016 | 17 | + | 50 | MH714667 |
| BACTEROIDETES | L-1968 | *Dyadobacter sp.* | Negative | 98 | Lake ice | Svalbard | 2016 | 10 | - | 125 | MN161208 |
|  | L-1981 | *Flavobacterium sp.* | Negative | 99 | Lake ice | Svalbard | 2016 | 10 | (+) | 100 | MN161213 |
|  | L-1994 | *Flavobacterium sp.* | Negative | 96 | Tap water | Svalbard | 2016 | 37 | + | 1000 | MN161215 |
|  | L-2291 | *Flavobacterium* sp. | Negative | 99 | Subglacial ice | Svalbard | 2017 | 15 | - | 5 | MK670552 |
|  | L-2560 | *Flavobacterium* sp. | Negative | 99 | Subglacial ice | Svalbard | 2017 | 15 | (-) | 5 | MK670522 |
|  | L-2562 | *Flavobacterium* sp. | Negative | 99 | Subglacial ice | Svalbard | 2017 | 15 | - | 10 | MK670519 |
|  | L-2563 | *Flavobacterium* sp. | Negative | 100 | Subglacial ice | Svalbard | 2017 | 15 | (+) | 20 | MK670520 |
|  | L-2573 | *Flavobacterium* sp. | Negative | 99 | Subglacial ice | Svalbard | 2017 | 15 | (-) | 5 | MK670541 |
|  | L-2662 | *Flavobacterium* sp. | Negative | 99 | Subglacial ice | Svalbard | 2017 | 15 | (-) | 5 | MK670523 |
|  | L-2564 | *Hymenobacter* sp. | Negative | 99 | Subglacial ice | Svalbard | 2017 | 15 | (-) | 5 | MK670531 |
|  | L-1969 | *Pedobacter sp.* | Negative | 99 | Lake ice | Svalbard | 2016 | 10 | (-) | 125 | MN161209 |
|  | L-1973 | *Pedobacter sp.* | Negative | 99 | Lake ice | Svalbard | 2016 | 10 | (-) | 50 | MN161211 |
|  | N36a (L-5696) | *Pedobacter sp.* | Negative | 99 | Sediment | Svalbard - Ny Alesund | 2017 | 17 | (+) | n/a | MH714626 |
| FIRMICUTES | 12396_bac | *Bacillus* sp. | Positive | 100 | Clear ice | Greenland | 2017 | 15 | + | 100 | MK453054 |
|  | 12403-Bac | *Bacillus* sp. | Positive | 100 | Dark ice | Greenland | 2017 | 15 | + | 0.1 | MK453055 |
|  | L-1896 | *Bacillus* sp. | Positive | 100 | Cryoconite | Greenland | 2016 | 15 | + | 0.1 | MK453101 |
|  | L-1906 | *Bacillus* sp. | Positive | 100 | Dispersed cryoconite | Greenland | 2016 | 15 | + | 0.1 | MK453109 |
|  | L-1907 | *Bacillus* sp. | Positive | 100 | Dispersed cryoconite | Greenland | 2016 | 15 | + | 0.1 | MK453110 |
|  | L-1910 | *Bacillus* sp. | Positive | 100 | Dispersed cryoconite | Greenland | 2016 | 15 | (+) | 0.1 | MK453112 |
|  | L-1917 | *Bacillus* sp. | Positive | 100 | Dispersed cryoconite | Greenland | 2016 | 15 | + | 0.1 | MK453119 |
|  | L-1921 | *Bacillus* sp. | Positive | 100 | Clear ice | Greenland | 2016 | 15 | + | 0.1 | MK453123 |
|  | S7 (L-5708) | *Bacillus* sp. | Positive | 99 | Sediment | Svalbard - Longyearbyen | 2016 | 37 | + | n/a | MH714653 |
|  | S44 (L-5716) | *Bacillus* sp. | Positive | 99 | Snow | Svalbard - Longyearbyen | 2016 | 37 | + | 50 | MH714670 |
|  | S70 (L-5719) | *Bacillus* sp. | Positive | 99 | Sediment | Svalbard - Longyearbyen | 2016 | 37 | + | n/a | MH714676 |
|  | S71 (L-5720) | *Bacillus* sp. | Positive | 99 | Sediment | Svalbard - Longyearbyen | 2016 | 37 | + | n/a | MH714677 |
|  | N23 (L-5692) | *Bacillus* sp. | Positive | 99 | Sediment | Svalbard - Ny Alesund | 2017 | 37 | (+) | n/a | MH714619 |
|  | N24 (L-5693) | *Bacillus* sp. | Positive | **83** | Sediment | Svalbard - Ny Alesund | 2017 | 37 | (+) | n/a | MH714620 |
|  | N34 (L-5695) | *Bacillus* sp. | Positive | 99 | Soil | Svalbard - Ny Alesund | 2017 | 37 | + | n/a | MH714624 |
|  | N83 (L-5705) | *Bacillus* sp. | Positive | 99 | Snow | Svalbard - Ny Alesund | 2017 | 17 | (+) | 3000 | MH714642 |
|  | S27b (L-5714) | Carnobacterium | Positive | 99 | Sediment | Svalbard - Longyearbyen | 2016 | 17 | (+) | n/a | MH714664 |
|  | N39 (L-5697) | Carnobacterium | Positive | 99 | Sediment | Svalbard - Ny Alesund | 2017 | 17 | (+) | n/a | MH714627 |
|  | N58 (L-5702) | Carnobacterium | Positive | 99 | Sediment | Svalbard - Ny Alesund | 2017 | 5 | - | n/a | MH714634 |
|  | An58 (L-5722) | Enterococcus | Positive | 99 | Sediment | Svalbard - Longyearbyen | 2016 | 17 | + | n/a | MH714608 |
|  | N2 (L-5689) | Exiguobacterium | Positive | 99 | Soil | Svalbard - Ny Alesund | 2017 | 37 | + | n/a | MH714612 |
|  | N7 (L-5690) | Paenibacillus | Positive | 99 | Soil | Svalbard - Ny Alesund | 2017 | 37 | + | n/a | MH714613 |
|  | L-2661 | *Paenibacillus* *antarcticus* | Positive | 100 | Subglacial ice | Svalbard | 2017 | 15 | - | 5 | MK670537 |
|  | N54 (L-5701) | Psychrobacillus | Positive | **94** | soil | Svalbard - Ny Alesund | 2017 | 37 | (+) | n/a | MH714632 |
| PROTEOBACTERIA | L-2655 | *Halomonas sp.* | Negative | 96 | Dark ice | Greenland | 2016 | 15 | (-) | 0.1 | MN161221 |
|  | L-2290 | *Herminiimonas* sp. | Negative | 99 | Subglacial ice | Svalbard | 2017 | 15 | (-) | 5 | MK670551 |
|  | L-2266 | *Janthinobacterium* sp. | Negative | 99 | Cryoconite | Greenland | 2017 | 15 | (+) | 10 | MK453066 |
|  | L-1995 | *Janthinobacterium sp.* | Negative | 99 | Tap water | Svalbard | 2016 | 37 | + | 1000 | MN161216 |
|  | L-2294 | *Janthinobacterium* sp. | Negative | 99 | Cryoconite | Greenland | 2017 | 15 | (-) | 10 | MK453067 |
|  | L-2271 | Massilia sp. | Negative | 100 | Dark ice | Greenland | 2016 | 15 | - | 0.1 | MN161219 |
|  | L-2283 | *Massilia* sp. | Negative | 99 | Subglacial ice | Svalbard | 2017 | 15 | - | 20 | MK670525 |
|  | L-2577 | *Massilia* sp. | Negative | 99 | Subglacial ice | Svalbard | 2017 | 15 | (-) | 20 | MK670545 |
|  | L-2653 | *Massilia* sp. | Negative | 99 | Glacial meltwater | Svalbard | 2017 | 15 | - | 10 | MK670506 |
|  | L-2275 | *Massilia* sp. | Negative | 99 | Supraglacial water | Greenland | 2017 | 15 | (-) | 100 | MK453068 |
|  | L-2276 | *Massilia* sp. | Negative | 99 | Supraglacial water | Greenland | 2017 | 15 | (-) | 100 | MK453069 |
|  | L-2558 | *Polaromonas* sp. | Negative | 99 | Glacial meltwater | Svalbard | 2017 | 15 | + | 10 | MK670510 |
|  | L-2565 | *Polaromonas* sp. | Negative | 100 | Subglacial ice | Svalbard | 2017 | 15 | (-) | 5 | MK670532 |
|  | N40 (L-5698) | Pseudomonas | Negative | 98 | Soil | Svalbard - Ny Alesund | 2017 | 5 | (+) | n/a | MN450717 |
|  | N71 (L-5704) | Pseudomonas | Negative | 99 | Soil | Svalbard - Ny Alesund | 2017 | 5 | + | n/a | MH714637 |
|  | L-2652 | *Pseudomonas frederiksbergensis* | Negative | 100 | Glacial meltwater | Svalbard | 2017 | 15 | (-) | 10 | MK670511 |
|  | L-2553 | *Pseudomonas graminis* | Negative | 100 | Glacial meltwater | Svalbard | 2017 | 15 | + | 10 | MK670514 |
|  | L-2646 | *Pseudomonas graminis* | Negative | 100 | Glacial meltwater | Svalbard | 2017 | 15 | (-) | 10 | MK670504 |
|  | L-1964 | *Pseudomonas sp.* | Negative | 99 | Lake ice | Svalbard | 2016 | 10 | - | 100 | MN161207 |
|  | L-1983 | *Pseudomonas sp.* | Negative | 99 | Lake ice | Svalbard | 2016 | 10 | (+) | 100 | MN161214 |
|  | L-2137 | *Pseudomonas* sp. | Negative | 100 | Cryoconite | Greenland | 2017 | 15 | + | 10 | MK453082 |
|  | L-2142 | *Pseudomonas* sp. | Negative | 99 | Cryoconite | Greenland | 2017 | 15 | (+) | 10 | MK453083 |
|  | L-2145 | *Pseudomonas* sp. | Negative | 100 | Cryoconite | Greenland | 2017 | 15 | + | 10 | MK453079 |
|  | L-2265 | *Pseudomonas* sp. | Negative | 100 | Cryoconite | Greenland | 2017 | 15 | + | 10 | MK453078 |
|  | L-2554 | *Pseudomonas* sp. | Negative | 100 | Glacial meltwater | Svalbard | 2017 | 15 | - | 10 | MK670515 |
|  | L-2575 | *Pseudomonas* sp. | Negative | 100 | Subglacial ice | Svalbard | 2017 | 15 | (-) | 5 | MK670547 |
|  | L-2578 | *Pseudomonas* sp. | Negative | 100 | Subglacial ice | Svalbard | 2017 | 15 | (-) | 20 | MK670546 |
|  | L-2643 | *Pseudomonas* sp. | Negative | 99 | Cryoconite | Greenland | 2017 | 15 | (-) | 10 | MK453084 |
|  | L-2644 | *Pseudomonas sp.* | Negative | 99 | Dark ice | Greenland | 2016 | 15 | (-) | 0.1 | MN161220 |
|  | L-2650 | *Pseudomonas* sp. | Negative | 99 | Glacial meltwater | Svalbard | 2017 | 15 | (-) | 50 | MK670512 |
|  | L-2656 | *Pseudomonas sp.* | Negative | 100 | Dark ice | Greenland | 2016 | 15 | (-) | 0.1 | MN161222 |
|  | L-2657 | *Pseudomonas sp.* | Negative | 99 | Dark ice | Greenland | 2016 | 15 | (+) | 0.1 | MN161223 |
|  | L-2658 | *Pseudomonas sp.* | Negative | 99 | Dark ice | Greenland | 2016 | 15 | (+) | 0.1 | MN161224 |
|  | L-2659 | *Pseudomonas sp.* | Negative | 99 | Dark ice | Greenland | 2016 | 15 | (+) | 0.1 | MN161225 |
|  | L-2696 | *Pseudomonas* sp. | Negative | 99 | Cryoconite | Greenland | 2017 | 15 | + | 10 | MK453081 |
|  | N61 (L-5703) | Psychrobacter | Negative | 99 | Sediment | Svalbard - Ny Alesund | 2017 | 17 | (-) | n/a | MH714636 |
|  | L-2695 | *Rahnella sp.* | Negative | 99 | Dark ice | Greenland | 2016 | 15 | (+) | 0.1 | MN161227 |
|  | L-1980 | *Raoultella sp.* | Negative | 99 | Lake ice | Svalbard | 2016 | 10 | + | 100 | MN161212 |
|  | L-1894 | *Rhodopseudomonas* sp. | Negative | 99 | Cryoconite | Greenland | 2016 | 15 | - | 0.1 | MK453099 |
|  | L-1900 | *Sphingomonas* sp. | Negative | 99 | Cryoconite | Greenland | 2016 | 15 | (-) | 0.1 | MK453105 |
|  | L-1972 | *Sphingomonas sp.* | Negative | 100 | Lake ice | Svalbard | 2016 | 10 | (-) | 50 | MN161210 |
|  | L-2273 | *Sphingomonas* sp. | Negative | 100 | Supraglacial water | Greenland | 2017 | 15 | (-) | 100 | MK453093 |
|  | L-2552 | *Sphingomonas* sp. | Negative | 100 | Cryoconite | Greenland | 2017 | 15 | - | 0.1 | MK453092 |
|  | L-2694 | *Sphingomonas sp.* | Negative | 99 | snow | Greenland | 2017 | 15 | + |  | MN161226 |
|  | L-2648 | *Sphingomonas* sp. (faeni) | Negative | 100 | Glacial meltwater | Svalbard | 2017 | 15 | (-) | 10 | MK670516 |
|  | L-2649 | *Sphingomonas* sp. (glacialis) | Negative | 100 | Glacial meltwater | Svalbard | 2017 | 15 | (-) | 10 | MK670517 |
|  | L-2651 | *Sphingorhabdus* sp. | Negative | 100 | Glacial meltwater | Svalbard | 2017 | 15 | (-) | 10 | MK670509 |
|  | L-2063 | *Undibacterium sp.* | Negative | 99 | Dark ice | Greenland | 2016 | 15 | (-) | 0.1 | MN161218 |
|  | L-2267 | *Undibacterium* sp. | Negative | 99 | Cryoconite | Greenland | 2017 | 15 | (-) | 0.1 | MK453095 |
|  | L-2647 | unidentified Burkholderiaceae | Negative | 99 | Glacial meltwater | Svalbard | 2017 | 15 | (-) | 10 | MK670505 |
|  | L-2432 | unidentified Oxalobacteraceae | Negative | 98 | Clear ice | Greenland | 2017 | 15 | (-) | 100 | MK453094 |
|  | L-2547 | *unidentified Oxalobacteraceae* | Negative | 98 | Supraglacial water | Greenland | 2017 | 15 | - | 0.1 | MK453072 |
|  | L-2146 | *unidentified Oxalobacteraceae* | Negative | 96 | Cryoconite | Greenland | 2017 | 15 | (-) | 0.1 | MK453089 |
|  | L-2270 | unidentified Oxalobacteraceae | Negative | 97 | Cryoconite | Greenland | 2017 | 15 | (-) | 0.1 | MK453071 |

**Table S3. List of the isolates selected for the antimicrobial susceptibility testing with the corresponding AMR profile. + indicates resistance; (+) indicates moderate (or reduced) resistance; - indicates susceptibility; (-) indicates moderate (or reduced) susceptibility; M indicates the appearance of individual mutant colonies.**

| **Isolate** | **Genus / Species** | **Growth** | **AMP** | **CHL** | **CIP** | **CTX** | **ERY** | **IPM** | **KAN** | **TET** |
| --- | --- | --- | --- | --- | --- | --- | --- | --- | --- | --- |
| L-1909 | *Arthrobacter* sp. | + | - | - | + | (-) | - | + | - | - |
| 12396_bac | *Bacillus* sp. | + | - | - | - | - | - | (-) | - | - |
| S7 | *Bacillus* sp. | + | + | - | - | + | - | + | - | - |
| S44 | *Bacillus* sp. | + | - | - | - | (-) | - | - | - | - |
| S70 | *Bacillus* sp. | + | (-) | - | - | + | - | + | - | - |
| S71 | *Bacillus* sp. | (-) | - | - | - | - | - | (-) | - | - |
| N23 | *Bacillus* sp. | (-) | - | - | - | (-) | + | - | - | - |
| N24 | *Bacillus* sp. | (+) | - | - | - | - | + | - | - | - |
| N34 | *Bacillus* sp. | + | - | - | - | - | - | - | - | - |
| N83 | *Bacillus* sp. | + | - | - | - | - | - | - | - | - |
| 12403-Bac | *Bacillus* sp. | + | - | - | - | - | - | + | - | - |
| L-1896 | *Bacillus* sp. | - | - | - | M | - | - | (+) | - | - |
| L-1906 | *Bacillus* sp. | - | - | - | + | - | - | + | - | - |
| L-1907 | *Bacillus* sp. | + | - | - | (-) | - | - | + | - | - |
| L-1910 | *Bacillus* sp. | + | - | - | - | - | - | - | - | - |
| L-1917 | *Bacillus* sp. | + | - | - | + | - | - | + | - | - |
| L-1921 | *Bacillus* sp. | + | - | - | + | - | - | + | - | - |
| S24 | *Brevibacterium* sp. | + | - | (+) | - | (-) | (+) | - | (-) | - |
| S27b | *Carnobacterium* sp. | (+) | - | - | - | + | - | (-) | (-) | - |
| N39 | *Carnobacterium* sp. | + | - | - | - | (-) | - | (-) | - | - |
| N58 | *Carnobacterium* sp. | - | - | - | - | - | - | - | - | - |
| L-1899 | *Cryobacterium psychrotolerans* sp. | (-) | - | - | - | - | - | + | - | - |
| L-2062 | *Cryobacterium* sp. | - | - | - | - | - | - | (-) | - | - |
| L-2263 | *Cryobacterium* sp. | (-) | - | - | (-) | - | - | (-) | - | - |
| L-2279 | *Cryobacterium* sp. | (-) | - | - | - | - | - | + | - | - |
| L-2285 | *Cryobacterium* sp. | - | - | - | - | - | - | - | - | - |
| L-2571 | *Cryobacterium* sp. | - | - | - | (-) | - | - | - | - | - |
| L-2580 | *Cryobacterium* sp. | - | - | - | - | - | - | (-) | - | - |
| L-2264 | *Curtobacterium* sp. | + | - | - | M | + | - | (+) | - | - |
| L-2433 | *Curtobacterium* sp. | + | - | - | (+) | (-) | - | (+) | - | - |
| L-1968 | *Dyadobacter* sp. | + | - | - | + | + | (-) | + | + | - |
| An58 | *Enterococcus* sp. | + | - | - | + | + | - | + | - | - |
| N2 | *Exiguobacterium* sp. | + | - | - | - | (-) | - | (+) | - | - |
| L-1981 | *Flavobacterium* sp. | + | - | - | + | + | (-) | + | + | - |
| L-1994 | *Flavobacterium* sp. | + | + | + | M | + | + | + | M | + |
| L-2562 | *Flavobacterium* sp. | + | - | - | (-) | + | (-) | + | + | - |
| L-2563 | *Flavobacterium* sp. | + | - | - | - | - | - | - | - | - |
| L-2430 | *Frigoribacterium* sp. | - | - | - | - | - | (-) | - | - | - |
| L-1995 | *Janthinobacterium* sp. | + | + | + | M | + | + | + | - | (-) |
| An34 | *Leifsonia* sp. | + | - | - | (-) | - | - | + | - | - |
| L-2271 | *Massilia* sp. | - | - | - | - | - | - | + | - | - |
| L-2283 | *Massilia* sp. | (+) | - | - | - | + | - | + | - | - |
| L-2577 | *Massilia* sp. | + | - | - | (-) | (+) | - | + | - | - |
| L-2653 | *Massilia* sp. | (+) | - | - | - | + | - | + | - | - |
| L-2275 | *Massilia* sp. | (-) | - | - | + | (-) | - | + | - | - |
| L-2276 | *Massilia* sp. | (-) | - | - | - | + | - | + | - | - |
| S8 | *Microbacterium* sp. | + | - | - | + | - | - | (+) | - | - |
| S23b | *Microbacterium* sp. | + | - | - | + | + | - | + | (-) | - |
| S26 | *Microbacterium* sp. | (+) | - | - | - | - | - | (-) | - | - |
| S60 | *Microbacterium* sp. | + | - | - | - | - | - | - | (+) | - |
| L-1922 | *Micrococcus* sp. sp. | (+) | - | - | (-) | - | (+) | + | - | - |
| S3 | *Micromonospora* sp. | (+) | (-) | - | (-) | + | + | - | (-) | - |
| N18 | *Micromonospora* sp. | + | - | (-) | - | + | (+) | - | (-) | - |
| S10 | *Oerskovia* sp. | + | - | - | (+) | + | (+) | + | (+) | (-) |
| N7 | *Paenibacillus* sp. | (-) | - | - | M | (-) | - | (+) | - | - |
| L-2661 | *Paenibacillus antarcticus* | + | - | - | + | - | - | + | - | - |
| N36a | *Pedobacter* sp. | + | - | - | + | + | + | + | + | - |
| L-1969 | *Pedobacter* sp. | + | + | - | + | + | (+) | + | + | - |
| L-1973 | *Pedobacter* sp. | + | + | - | + | + | - | + | + | - |
| L-2558 | *Polaromonas* sp. | + | - | - | + | + | - | + | - | - |
| L-2565 | *Polaromonas* sp. | (+) | - | - | - | - | - | (+) | - | - |
| N40 | *Pseudomonas* sp. | + | + | + | M | + | + | + | - | - |
| N71 | *Pseudomonas* sp. | + | + | + | M | + | + | + | (-) | - |
| L-2652 | *Pseudomonas frederiksbergensis* | + | + | + | M | + | + | + | - | - |
| L-2553 | *Pseudomonas graminis* | + | - | - | - | + | - | + | - | - |
| L-2646 | *Pseudomonas graminis* | + | - | (-) | - | + | - | + | - | - |
| L-1964 | *Pseudomonas* sp. | + | + | + | - | + | + | + | - | (-) |
| L-1983 | *Pseudomonas* sp. | + | + | + | M | + | + | + | - | (-) |
| L-2137 | *Pseudomonas* sp. | + | + | + | M | + | + | + | - | (-) |
| L-2142 | *Pseudomonas* sp. | + | + | + | M | + | + | + | - | (-) |
| L-2145 | *Pseudomonas* sp. | + | + | + | M | + | + | + | - | - |
| L-2265 | *Pseudomonas* sp. | + | + | + | M | + | + | + | - | - |
| L-2554 | *Pseudomonas* sp. | + | + | + | M | + | + | + | - | - |
| L-2575 | *Pseudomonas* sp. | + | + | + | M | + | + | + | - | - |
| L-2578 | *Pseudomonas* sp. | + | + | + | M | + | + | + | - | - |
| L-2643 | *Pseudomonas* sp. | + | + | + | M | + | + | + | - | (-) |
| L-2644 | *Pseudomonas fluorescens* | + | + | + | M | + | + | + | (-) | - |
| L-2650 | *Pseudomonas* sp. | + | + | + | M | + | + | + | - | - |
| L-2656 | *Pseudomonas fluorescens* | + | + | + | M | + | + | + | (-) | - |
| L-2657 | *Pseudomonas* sp. | + | + | + | M | + | + | + | - | - |
| L-2658 | *Pseudomonas fluorescens* | + | + | + | M | + | + | + | (-) | - |
| L-2659 | *Pseudomonas* sp. | + | + | + | M | + | + | + | - | - |
| L-2696 | *Pseudomonas* sp. | + | + | + | (+) | + | + | + | - | - |
| N54 | *Psychrobacillus* sp. | - | - | - | - | - | - | - | - | - |
| N61 | *Psychrobacter* sp. | + | - | - | - | - | - | + | - | - |
| L-2695 | *Rahnella* sp. | + | + | - | - | (+) | (-) | + | - | + |
| L-1980 | *Raoultella* sp. | + | (+) | - | - | - | (+) | + | - | - |
| L-1894 | *Rhodopseudomonas* sp. | - | - | - | - | - | - | (-) | - | - |
| S58 | *Salinibacterium* sp. | (-) | - | - | - | - | - | - | (+) | - |
| N106 | *Salinibacterium* sp. | - | - | - | - | - | - | - | - | - |
| L-1900 | *Sphingomonas* sp. | - | - | - | (-) | - | - | (-) | - | - |
| L-1972 | *Sphingomonas* sp. | + | - | - | - | + | - | + | - | - |
| L-2273 | *Sphingomonas* sp. | - | - | - | (+) | - | - | (-) | - | - |
| L-2694 | *Sphingomonas* sp. | + | - | - | - | + | (-) | + | - | - |
| L-2648 | *Sphingomonas* sp. *(faeni)* | + | - | - | - | + | - | + | - | - |
| L-2649 | *Sphingomonas* sp. *(glacialis)* | + | - | - | + | (+) | - | (+) | - | - |
| N28 | *Streptomyces* sp. | (+) | - | - | M | (+) | - | - | - | - |
| N41 | *Streptomyces* sp. | + | + | (-) | (-) | + | - | + | - | - |
| N42 | *Streptomyces* sp. | (+) | - | - | (+) | + | + | - | - | - |
| S32 | *Tessaracoccus* sp. | (+) | - | - | - | (+) | (+) | - | (+) | - |
| L-2063 | *Undibacterium* sp. | - | - | - | (-) | - | - | (+) | - | - |
| L-2267 | *Undibacterium* sp. | - | - | - | - | - | - | (-) | - | - |
| L-2647 | unidentified Burkholderiaceae | + | - | (-) | + | + | - | + | - | - |
| L-2146 | unidentified Oxalobacteraceae | - | - | - | - | - | - | (-) | - | - |

**Table S4. List of the isolates with imipenem resistance further inoculated on: 1. Nutrient agar plates containing 4, 6, 8 and 10mg/l of imipenem and 2. CHROMID® CARBA SMART Agar, for the detection of carbapenemases. In red text, those isolates that were positive for both types of carbapenemases screened in the agar. O: OXA-48 Carbapenemase; C: carbapenemases other than OXA-48 such as *Klebsiella pneumoniae* carbapenemases (KPC) or metallo-β-lactamases (MBL) including New Delhi metallo-β-lactamases (NDM); + indicates resistance; (+) indicates moderate (or reduced) resistance; - indicates susceptibility; (-) indicates moderate (or reduced) susceptibility; NT: not tested**

| **Isolate** | **Genus / Species** | **IPM (4mg/l)** | **IMP resistance range (4-10mg/l)** | **Phenotype for IPM resistance range** | **Phenotype on CHROMID® CARBA SMART Agar** |
| --- | --- | --- | --- | --- | --- |
| L-1909 | *Arthrobacter* sp. | + | 4 mg | (-) | NT |
| S70 (L-5719) | *Bacillus wiedmannii* | + | 4 mg | (+) | C-,O- |
| S7 (L-5708) | *Bacillus cereus* | + | 4 mg | (-) | NT |
| 12403-Bac | *Bacillus* sp. | + | 4 mg | (-) | NT |
| L-1906 | *Bacillus sp. (acidiceler / luciferensis)* | + | 4 mg | (-) | NT |
| L-1907 | *Bacillus sp. (acidiceler / luciferensis)* | + | 4 mg | (-) | NT |
| L-1917 | *Bacillus sp. (acidiceler / luciferensis)* | + | 4 mg | (-) | NT |
| L-1921 | *Bacillus sp. (acidiceler / luciferensis)* | + | 4 mg | (-) | NT |
| L-1899 | *Cryobacterium psychrotolerans* | + | 4 mg | (-) | C-,O- |
| L-2264 | *Curtobacterium* sp. | (+) | 4 mg | (-) | NT |
| L-2433 | *Curtobacterium* sp. | (+) | 4 mg | (-) | NT |
| L-1968 | *Dyadobacter sp. DR5 Antarctica* | + | 4 mg | (-) | NT |
| An58 (L-5722) | *Enterococcus faecium* | + | 4-(6) mg | (+) | C-,O- |
| N2 (L-5689) | *Exiguobacterium undae* | (+) | 4 mg | (-) | NT |
| L-1994 | *Flavobacterium* sp. | + | 4-10 mg | (+) | **C+,O+** |
| L-2562 | *Flavobacterium* sp. | + | 4-6 mg | (+) | NT |
| L-1981 | *Flavobacterium sp. CV41May* | + | 4-10 mg | (+) | C-,O- |
| L-1995 | *Janthinobacterium* sp. | + | 4 mg | (+) | **C+,O+** |
| An34 (L-5721) | *Leifsonia kafniensis kafniensis* | + | 4 mg | (-) | NT |
| S23b (L-5711) | *Microbacterium maritypicum* | + | 4 mg | (+) | C-,O- |
| S8 (L-5709) | *Microbacterium lacticum* | (+) | 4 mg | (-) | C-,O- |
| L-1922 | *Micrococcus lactis* | + | 4 mg | (-) | C-,O- |
| S10 (L-5710) | *Oerskovia paurometabola* | + | 4 mg | (-) | NT |
| N36a (L-5696) | *Pedobacter nyackensis* | + | 4 mg | (-) | NT |
| L-1969 | *Pedobacter sp. JR29* | + | 4 mg | (-) | NT |
| L-2558 | *Polaromonas* sp. | + | 4 mg | (-) | NT |
| N40 (L-5698) | *Pseudomonas helmanticensis* | + | 4 mg | (-) | NT |
| N71 (L-5704) | *Pseudomonas lurida* | + | 4-10 mg | (+) | **C+,O+** |
| L-2644 | *Pseudomonas fluorescens* | + | 4-10 mg | (+) | **C+,O+** |
| L-2656 | *Pseudomonas fluorescens* | + | 4-10 mg | (+) | **C+,O+** |
| L-2658 | *Pseudomonas fluorescens* | + | 4-10 mg | (+) | **C+,O+** |
| L-1983 | *Pseudomonas fluorescens strain TML16* | + | 4-6 mg | (+) | **C+,O+** |
| L-2652 | *Pseudomonas frederiksbergensis* | + | 4 mg | (-) | NT |
| L-2553 | *Pseudomonas graminis* | + | 4 mg | (-) | NT |
| L-2646 | *Pseudomonas graminis* | + | 4 mg | (-) | NT |
| L-2137 | *Pseudomonas* sp. | + | 4-10 mg | (+) | **C+,O+** |
| L-2142 | *Pseudomonas* sp. | + | 4-10 mg | (+) | **C+,O+** |
| L-2145 | *Pseudomonas* sp. | + | 4-10 mg | (+) | **C+,O+** |
| L-2265 | *Pseudomonas* sp. | + | 4-10 mg | (+) | **C+,O+** |
| L-2554 | *Pseudomonas* sp. | + | 4-6 mg | (+) | **C+,O+** |
| L-2575 | *Pseudomonas* sp. | + | 4 mg | (+) | **C+,O+** |
| L-2578 | *Pseudomonas* sp. | + | 4 mg | (-) | **C+,O+** |
| L-2643 | *Pseudomonas* sp. | + | 4-10 mg | (+) | **C+,O+** |
| L-2650 | *Pseudomonas* sp. | + | 4-10 mg | (+) | **C+,O+** |
| L-2657 | *Pseudomonas* sp. | + | 4-8 mg | (+) | NT |
| L-2659 | *Pseudomonas* sp. | + | 4-10 mg | (+) | NT |
| L-2696 | *Pseudomonas* sp. | + | 4-10 mg | (+) | C-,O- |
| L-1964 | *Pseudomonas sp. (yamanorum or KP-1-4)* | + | 4 mg | (-) | NT |
| 61 (NL-5703) | *Psychrobacter nivimaris* | + | 4 mg | (-) | NT |
| L-2695 | *Rahnella* sp. | + | 4 mg | (-) | C-,O+ |
| L-1980 | *Raoultella ornithinolytica strain FDAARGOS_431* | + | 4 mg | (+) | **C+,O+** |
| L-1972 | *Sphingomonas* sp. | + | 4 mg | (-) | C-,O- |
| L-2694 | *Sphingomonas* sp. | + | 4 mg | (-) | C-,O- |
| L-2648 | *Sphingomonas* sp. *(faeni)* | + | 4 mg | (-) | C-,O- |
| N41 (L-5699) | *Streptomyces candidus* | + | 4 mg | (-) | NT |
| N28 (L-5694) | *Streptomyces avidinii* | - | 4 mg | (+) | C-,O- |
| L-2647 | unidentified Burkholderiaceae | + | 4 mg | (-) | C-,O- |


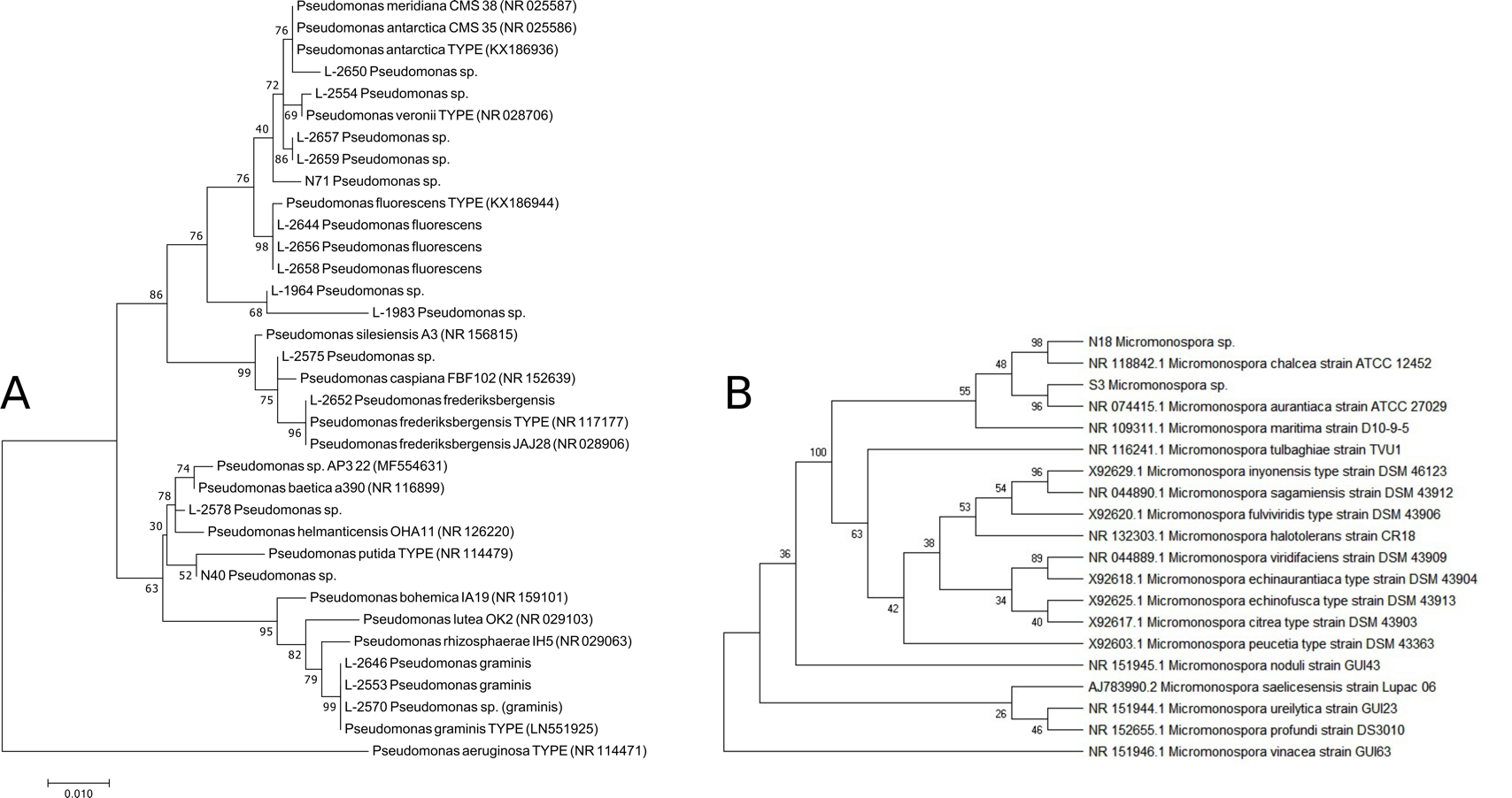


**Figure S1** Phylogenetic analyses of the 16S sequences of Pseudomonas species (A) and Micromonospora species (B) from Arctic environments compared with sequences of type strains or other closest species deposited in the GenBank database. The tree was constructed using the maximum composite likelihood method. Bootstrap values are shown (500 replicates). The sequence of Pseudomonas aeruginosa (NR_114471) (A) and Micromonospora vinacea (NR_151946) (B) were used as outgroups, respectively. Evolutionary analyses were conducted in MEGA 7.
